# Variant-specific changes in RAC3 function disrupt corticogenesis in neurodevelopmental phenotypes

**DOI:** 10.1093/brain/awac106

**Published:** 2022-07-04

**Authors:** Marcello Scala, Masashi Nishikawa, Hidenori Ito, Hidenori Tabata, Tayyaba Khan, Andrea Accogli, Laura Davids, Anna Ruiz, Pietro Chiurazzi, Gabriella Cericola, Björn Schulte, Kristin G Monaghan, Amber Begtrup, Annalaura Torella, Michele Pinelli, Anne Sophie Denommé-Pichon, Antonio Vitobello, Caroline Racine, Maria Margherita Mancardi, Courtney Kiss, Andrea Guerin, Wendy Wu, Elisabeth Gabau Vila, Bryan C Mak, Julian A Martinez-Agosto, Michael B Gorin, Bugrahan Duz, Yavuz Bayram, Claudia M B Carvalho, Jaime E Vengoechea, David Chitayat, Tiong Yang Tan, Bert Callewaert, Bernd Kruse, Lynne M Bird, Laurence Faivre, Marcella Zollino, Saskia Biskup, Undiagnosed Diseases Network, Gabrielle Brown, Manish J Butte, Esteban C Dell'Angelica, Naghmeh Dorrani, Emilie D Douine, Brent L Fogel, Irma Gutierrez, Alden Huang, Deborah Krakow, Hane Lee, Sandra K Loo, Bryan C Mak, Martin G Martin, Julian A Martínez-Agosto, Elisabeth McGee, Stanley F Nelson, Shirley Nieves-Rodriguez, Christina G S Palmer, Jeanette C Papp, Neil H Parker, Genecee Renteria, Janet S Sinsheimer, Jijun Wan, Lee-kai Wang, Katherine Wesseling Perry, Telethon Undiagnosed Diseases Program, Vincenzo Nigro, Nicola Brunetti-Pierri, Giorgio Casari, Gerarda Cappuccio, Annalaura Torella, Michele Pinelli, Francesco Musacchia, Margherita Mutarelli, Diego Carrella, Giuseppina Vitiello, Valeria Capra, Giancarlo Parenti, Vincenzo Leuzzi, Angelo Selicorni, Silvia Maitz, Sandro Banfi, Marcella Zollino, Mario Montomoli, Donatelli Milani, Corrado Romano, Albina Tummolo, Daniele De Brasi, Antonietta Coppola, Claudia Santoro, Angela Peron, Chiara Pantaleoni, Raffaele Castello, Stefano D’Arrigo, Pasquale Striano, Vincenzo Nigro, Mariasavina Severino, Valeria Capra, Gregory Costain, Koh ichi Nagata

**Affiliations:** Department of Neurosciences, Rehabilitation, Ophthalmology, Genetics, Maternal and Child Health, University of Genoa, Genoa, Italy; Pediatric Neurology and Muscular Diseases Unit, IRCCS Istituto Giannina Gaslini, Genoa, Italy; Department of Molecular Neurobiology, Institute for Developmental Research, Aichi Developmental Disability Center, 713-8 Kamiya, Kasugai 480-0392, Japan; Department of Molecular Neurobiology, Institute for Developmental Research, Aichi Developmental Disability Center, 713-8 Kamiya, Kasugai 480-0392, Japan; Department of Molecular Neurobiology, Institute for Developmental Research, Aichi Developmental Disability Center, 713-8 Kamiya, Kasugai 480-0392, Japan; Department of Molecular Neurobiology, Institute for Developmental Research, Aichi Developmental Disability Center, 713-8 Kamiya, Kasugai 480-0392, Japan; Genetics and Genome Biology, Research Institute, The Hospital for Sick Children, Toronto, Ontario, Canada; Department of Neurosciences, Rehabilitation, Ophthalmology, Genetics, Maternal and Child Health, University of Genoa, Genoa, Italy; Department of Human Genetics, Emory Healthcare, Atlanta, GA 30322, USA; Genetics Laboratory, UDIAT-Centre Diagnòstic, Parc Taulí Hospital Universitari, Institut d'Investigació i Innovació Parc Taulí I3PT, Universitat Autònoma de, Barcelona, Sabadell, Spain; Dipartimento Universitario Scienze della Vita e Sanità Pubblica, Sezione di Medicina Genomica, Università Cattolica Sacro Cuore, Rome, Italy; Genetica Medica, Fondazione Policlinico Universitario A. Gemelli IRCCS, Rome, Italy; Neuropediatric Department, Helios-Klinikum Hildesheim, Hildesheim, Germany; Praxis für Humangenetik, Tübingen, Germany; GeneDx, Gaithersburg, MD, USA; GeneDx, Gaithersburg, MD, USA; Telethon Institute of Genetics and Medicine, Pozzuoli, Naples, Italy; Department of Precision Medicine, University of Campania Luigi Vanvitelli, Naples, Italy; Telethon Institute of Genetics and Medicine, Pozzuoli, Naples, Italy; INSERM UMR1231 Génétique des Anomalies du Développement, Université de Bourgogne Franche-Comté, Dijon, France; Laboratoire de Génétique Moléculaire, UF Innovation en diagnostic génomique des maladies rares, Plateau Technique de Biologie, CHU de Dijon Bourgogne, Dijon, France; Centre de Génétique et Centre de Référence Anomalies du Développement et Syndromes Malformatifs de l'interrégion Est, FHU TRANSLAD, Hôpital d'Enfants, CHU de Dijon Bourgogne, Dijon, France; INSERM UMR1231 Génétique des Anomalies du Développement, Université de Bourgogne Franche-Comté, Dijon, France; Laboratoire de Génétique Moléculaire, UF Innovation en diagnostic génomique des maladies rares, Plateau Technique de Biologie, CHU de Dijon Bourgogne, Dijon, France; Laboratoire de Génétique Moléculaire, UF Innovation en diagnostic génomique des maladies rares, Plateau Technique de Biologie, CHU de Dijon Bourgogne, Dijon, France; Centre de Génétique et Centre de Référence Anomalies du Développement et Syndromes Malformatifs de l'interrégion Est, FHU TRANSLAD, Hôpital d'Enfants, CHU de Dijon Bourgogne, Dijon, France; Unit of Child Neuropsychiatry, Department of Medical and Surgical Neuroscience and Rehabilitation, IRCCS Istituto Giannina Gaslini, Genova, Italy; Division of Medical Genetics, Department of Pediatrics, Queen’s University, Kingston, ON K7L 2V7, Canada; Division of Medical Genetics, Department of Pediatrics, Queen’s University, Kingston, ON K7L 2V7, Canada; Genetics and Genome Biology, Research Institute, The Hospital for Sick Children, Toronto, Ontario, Canada; Queen’s University, Kingston, ON, Canada; Paediatric Unit, Parc Taulí Hospital Universitari, Institut d'Investigació i Innovació Parc Taulí I3PT, Universitat Autònoma de, Barcelona, Sabadell, Spain; Department of Human Genetics, David Geffen School of Medicine, University of California-Los Angeles, Los Angeles, CA, USA; Department of Human Genetics, David Geffen School of Medicine, University of California-Los Angeles, Los Angeles, CA, USA; Department of Pediatrics, David Geffen School of Medicine, University of California-Los Angeles, Los Angeles, CA, USA; Department of Psychiatry and Biobehavioral Sciences, David Geffen School of Medicine, University of California-Los Angeles, Los Angeles, CA, USA; Department of Human Genetics, David Geffen School of Medicine, University of California-Los Angeles, Los Angeles, CA, USA; Department of Ophthalmology, Jules Stein Eye Institute, David Geffen School of Medicine, UCLA, Los Angeles 90095, CA, USA; Brain Research Institute, UCLA, Los Angeles 90095, CA, USA; Haseki Training and Research Hospital, Istanbul, Turkey; Division of Genomic Diagnostics, Department of Pathology and Laboratory Medicine, Children’s Hospital of Philadelphia, Philadelphia, PA 19104, USA; Perelman School of Medicine, University of Pennsylvania, Philadelphia, PA 19104, USA; Pacific Northwest Research Institute, Seattle, WA 98122, USA; Baylor College of Medicine, Houston, TX 77030, USA; Department of Human Genetics, Emory Healthcare, Atlanta, GA 30322, USA; The Prenatal Diagnosis and Medical Genetics Program, Department of Obstetrics and Gynecology, Mount Sinai Hospital, University of Toronto, Toronto, Ontario, Canada; Division of Clinical and Metabolic Genetics, Department of Paediatrics, The Hospital for Sick Children, Toronto, Ontario, Canada; Department of Molecular Genetics, University of Toronto, Toronto, Ontario, Canada; Victorian Clinical Genetics Services, Murdoch Children’s Research Institute, and Department of Paediatrics, University of Melbourne, Melbourne, VIC 3052, Australia; Center for Medical Genetics, Ghent University Hospital, Gent, Belgium; Neuropediatric Department, Helios-Klinikum Hildesheim, Hildesheim, Germany; Department of Pediatrics, University of California San Diego, San Diego, CA, USA; Genetics/Dysmorphology, Rady Children’s Hospital San Diego, San Diego, CA, USA; INSERM UMR1231 Génétique des Anomalies du Développement, Université de Bourgogne Franche-Comté, Dijon, France; Centre de Génétique et Centre de Référence Anomalies du Développement et Syndromes Malformatifs de l'interrégion Est, FHU TRANSLAD, Hôpital d'Enfants, CHU de Dijon Bourgogne, Dijon, France; Dipartimento Universitario Scienze della Vita e Sanità Pubblica, Sezione di Medicina Genomica, Università Cattolica Sacro Cuore, Rome, Italy; Genetica Medica, Fondazione Policlinico Universitario A. Gemelli IRCCS, Rome, Italy; Praxis für Humangenetik, Tübingen, Germany; CeGaT GmbH, Tübingen, Germany; Department of Neurosciences, Rehabilitation, Ophthalmology, Genetics, Maternal and Child Health, University of Genoa, Genoa, Italy; Pediatric Neurology and Muscular Diseases Unit, IRCCS Istituto Giannina Gaslini, Genoa, Italy; Telethon Institute of Genetics and Medicine, Pozzuoli, Naples, Italy; Department of Precision Medicine, University of Campania Luigi Vanvitelli, Naples, Italy; Neuroradiology Unit, IRCCS Istituto Giannina Gaslini, Genoa, Italy; Medical Genetics Unit, IRCCS Istituto Giannina Gaslini, Genoa, Italy; Genetics and Genome Biology, Research Institute, The Hospital for Sick Children, Toronto, Ontario, Canada; Division of Clinical and Metabolic Genetics, Department of Paediatrics, The Hospital for Sick Children, Toronto, Ontario, Canada; Department of Molecular Genetics, University of Toronto, Toronto, Ontario, Canada; Department of Paediatrics, University of Toronto, Toronto, Ontario, Canada; Department of Molecular Neurobiology, Institute for Developmental Research, Aichi Developmental Disability Center, 713-8 Kamiya, Kasugai 480-0392, Japan; Department of Neurochemistry, Nagoya University Graduate School of Medicine, 65 Tsurumai-cho, Nagoya 466-8550, Japan

**Keywords:** *RAC3*, small GTPase, brain development, axon guidance, neuronal migration

## Abstract

Variants in *RAC3*, encoding a small GTPase RAC3 which is critical for the regulation of actin cytoskeleton and intracellular signal transduction, are associated with a rare neurodevelopmental disorder with structural brain anomalies and facial dysmorphism.

We investigated a cohort of 10 unrelated participants presenting with global psychomotor delay, hypotonia, behavioural disturbances, stereotyped movements, dysmorphic features, seizures and musculoskeletal abnormalities. MRI of brain revealed a complex pattern of variable brain malformations, including callosal abnormalities, white matter thinning, grey matter heterotopia, polymicrogyria/dysgyria, brainstem anomalies and cerebellar dysplasia. These patients harboured eight distinct *de novo RAC3* variants, including six novel variants (NM_005052.3): c.34G > C p.G12R, c.179G > A p.G60D, c.186_188delGGA p.E62del, c.187G > A p.D63N, c.191A > G p.Y64C and c.348G > C p.K116N. We then examined the pathophysiological significance of these novel and previously reported pathogenic variants p.P29L, p.P34R, p.A59G, p.Q61L and p.E62K. *In vitro* analyses revealed that all tested RAC3 variants were biochemically and biologically active to variable extent, and exhibited a spectrum of different affinities to downstream effectors including p21-activated kinase 1. We then focused on the four variants p.Q61L, p.E62del, p.D63N and p.Y64C in the Switch II region, which is essential for the biochemical activity of small GTPases and also a variation hot spot common to other Rho family genes, *RAC1* and *CDC42*. Acute expression of the four variants in embryonic mouse brain using *in utero* electroporation caused defects in cortical neuron morphology and migration ending up with cluster formation during corticogenesis. Notably, defective migration by p.E62del, p.D63N and p.Y64C were rescued by a dominant negative version of p21-activated kinase 1.

Our results indicate that *RAC3* variants result in morphological and functional defects in cortical neurons during brain development through variant-specific mechanisms, eventually leading to heterogeneous neurodevelopmental phenotypes.

## Teaser

*Scala et al*. identify six de novo variants in *RAC3*, which encodes a small GTPase, in 10 unrelated subjects with neurodevelopmental phenotypes. *In vivo* and *in vitro* analyses in mice reveal that *RAC3* variants cause morpho-functional defects in cortical neurons through variant-specific mechanisms, disrupting corticogenesis.

## Introduction

Rho family guanosine triphosphatases (GTPases) are key regulators of cellular signalling and cytoskeletal dynamics, which influence cell adhesion, morphology, migration and cell cycle progression, and play crucial roles in various cell types.^1^ The Rac subfamily consists of three proteins, Rac1–3, which share about 90% identity in their amino acid (aa) sequences, with the largest degree of divergence at their carboxy-terminal ends. While Rac1 is ubiquitously expressed in most organs^2^ and Rac2 is specifically expressed in hematopoietic cells,^3^ Rac3 is abundantly expressed both in the developing and adult nervous system.^4,5^ Accordingly, Rac3 has been reported to participate in different aspects of neuronal development, such as neuritogenesis, axon and dendrites formation, synaptogenesis and regulation of migration.^6–10^ Like other small GTPases, Rac3 is thought to act as a molecular switch cycling between GTP-bound (active) and GDP-bound (inactive) states via conformational changes in the Switch I and Switch II regions, which are highly conserved in small GTPases. The Switch II region determines the selective interaction with functionally diverse guanine nucleotide-exchange factors (GEFs) and GTPase-activating proteins (GAPs), which are the major classes of regulators controlling the guanine nucleotide-binding state.^11^ GEFs promote GTP-loading to activate small GTPases, while GAPs stimulate intrinsic GTP-hydrolysis to inactivate them. In addition to GEFs and GAPs, the Switch I region interacts with downstream effector molecules whose activities are strictly regulated in a spatiotemporal manner by activated small GTPases. Although Rac1 and Rac3 share common downstream effectors, their distinct expression profiles strongly suggest isoform-specific functions.

Accumulating evidence supports critical roles for Rho family GTPases in neural development,^1,12,13^ and indicates that the disruption of Rho GTPase signalling significantly contributes to the pathogenesis of neurodevelopmental disorders (NDDs) in humans.^14,15^ In the Simons Foundation Autism Research Initiative (SFARI) database, 7/82 RhoGEFs (9%), 7/57 RhoGAPs (12%), and 6/73 downstream effectors (8%) are categorized as autism spectrum disorders (ASD) candidate genes.^14^ At least 1.6% of all 1231 listed ASD candidate genes are therefore directly involved in the signalling pathways of Rho family GTPases. More specifically, the link between the abnormal function of Rho GTPases *per se* and NDDs has been recently highlighted as follows. As for the Rac subfamily, *de novo* missense *RAC1* variants affecting protein function have been associated with heterogeneous conditions characterized by variable psychomotor delays and brain malformations,^16^ while variations in *RAC2* have been identified in patients with different forms of primary immunodeficiencies.^17^ Recently, a novel NDD with structural brain anomalies and dysmorphic facies (NEDBAF, OMIM 618577) has been reportedly in association with *RAC3* variations.^18,19^ This condition is characterized by global developmental delay, intellectual disability, abnormal muscle tone, heterogeneous neurological phenotypes and structural brain abnormalities.^18,19^ Although the reported variants appear to hamper RAC3’s basic functions, such as actin cytoskeletal organization and signal transduction, the exact molecular mechanisms involved in the pathogenesis of NEDBAF remain to be elucidated.

In the present study, we investigated a cohort of 10 unrelated participants presenting with a syndromic NDD and peculiar brain malformations, including one partially reported case.^20^ We identified six novel *de novo* variants in *RAC3*, including five missense and one in-frame deletion. *In vitro* analyses revealed that the novel and previously reported 11 variants were biochemically and biologically active to variable extent and seemed to interact with various downstream effectors in variant-specific manners. *In vivo* analyses revealed that the four variants in the Switch II region caused morphological and migration defects of excitatory neurons during corticogenesis, with the involvement of a functional dysregulation of the downstream effector PAK1 in three out of four cases. In addition, the four variants prevented axonal development *in vivo*. Collectively, RAC3 was found to play a crucial role in brain development and pathogenic variants affecting its function are most likely to cause impaired corticogenesis, leading to the neurodevelopmental phenotypes observed in patients with NEDBAF associated with *RAC3* abnormalities.

## Materials and methods

### Ethics statement

The study involving human participants was conducted according to the guidelines of the Declaration of Helsinki. Ethical review and approval were obtained locally and informed consent was obtained from the parents or legal guardians of all the enrolled participants. For the animal experiments, we followed the fundamental guidelines for proper conduct of animal experiments and related activity in academic research institutions under the jurisdiction of the Ministry of Education, Culture, Sports, Science, and Technology (Japan). All protocols for animal handling and treatment were reviewed and approved by the Animal Care and Use Committee of Institute for Developmental Research, Aichi Developmental Disability Center (approval number: 2019-013).

### Patient enrolment and ascertainment

Patients were recruited through international collaboration, also using GeneMatcher,^21^ from several clinical and research centres in Canada, France, Germany, Italy, Spain and the USA (further details available in the Supplementary material). Patients were evaluated by paediatricians with expertise in neurological disorders, paediatric neurologists and geneticists with expertise in the field of neurogenetics. Vineland Adaptive Behavior Scale (VABS, third edition) tests were also administered for adaptive behaviour assessment to Patients 4, 5 and 8. Clinical and molecular data of Patient 6, who was partially described in a previous report,^20^ were critically reviewed. Brain MRI scans, locally performed for patient care, were reviewed by an expert paediatric neuroradiologist (M.S.). Previously published neuroimaging studies were also thoroughly reviewed for comparison and delineation of the neuroradiological spectrum.

### Previously reported cases assessment

All the articles indexed in PubMed (https://pubmed.ncbi.nlm.nih.gov/?term=RAC3&sort=date) between April 2019, when *RAC3* variants were first associated with NEDBAF by Costain *et al*.,^18^ and May 2021^22^ were retrieved using the terms ‘RAC3’, ‘Rac3 GTPase’ and ‘neurodevelopmental syndrome’. All the articles concerning the molecular, clinical, and neuroradiological spectrum associated with NEDBAF were thoroughly reviewed. Inclusion criteria for previously published patients included clinical data availability, unambiguous identification of pathogenic/likely pathogenic *RAC3* variants and lack of duplication from other previous reports. Ambiguous clinical presentation not consistent with NEDBAF and inconclusive genetic testing were exclusion criteria.

### Next generation sequencing

We investigated 10 individuals presenting with syndromic NDD and neuroimaging abnormalities. Genomic DNA was extracted from peripheral blood leucocytes using standard protocols (Supplementary material). Different next generation sequencing (NGS) approaches were employed, including trio-exome sequencing (Trio-ES) (Patients 1–3, 8 and 10), singleton exome sequencing (Patients 4, 6 and 9), trio-genome sequencing and RNA sequencing (Patient 5) and a NGS panel including 480 intellectual disability genes (Patient 7). Sanger sequencing was performed for the validation of the candidate variants and the parental segregation analysis. The presence of copy number variants was investigated in all subjects, either through array comparative genomic hybridization (aCGH) (Patients 1, 2, 4, 5, 6, 7, 8, 9 and 10) or CNV detection on exome sequencing data (Patient 3) (Supplementary material).

### Variant identification and analysis

The identified variants were filtered according to minor allele frequency ≤0.001 in Genome Aggregation Database (gnomAD, https://gnomad.broadinstitute.org),^23^ conservation (Genomic Evolutionary Rate Profiling—GERP, http://mendel.stanford.edu/SidowLab/downloads/gerp/) and predicted impact on protein function. *In silico* prediction tools were used for the interpretation of candidate variants, including combined annotation dependent depletion (CADD, https://cadd.gs.washington.edu), Mutation Taster (http://www.mutationtaster.org), Sorting Intolerant From Tolerant (SIFT, https://sift.bii.a-star.edu.sg) and Polyphen-2 (http://genetics.bwh.harvard.edu/pph2/). Ultimately, the American College of Medical Genetics and Genomics and the Association for Molecular Pathology (ACMG-AMP) guidelines were used to classify the candidate variants based on their predicted pathogenicity.^24^ All *RAC3* variants are reported according to the NM_005052.3 transcript (NP_005043.1) (https://www.ncbi.nlm.nih.gov/nuccore/NM_005052.3). Further details are available in the Supplementary material.

### Plasmids

*RAC3* was amplified by PCR from a cDNA pool of human glioblastoma U251MG cells and cloned into the pCAG-Myc vector. The 11 variants, *RAC3*-G12R, -P29L, -P34R, -A59G, -G60D, -Q61L, -E62del, -E62K, -D63N, -Y64C and -K116N, were generated by site-directed mutagenesis using KOD-Plus Mutagenesis kit (Toyobo Inc) with pCAG-Myc-RAC3 as a template. *RAC3* and the 11 variants were also constructed into pTriEx-4 vector (Merck). pRK5-Myc-PAK1 (p21-activated kinase 1)-KA, a kinase-negative variant with a single amino acid substitution p.K299A and pRK5-Myc-MLK2 (mixed lineage kinase 2/MAPKKK10)-KN, a kinase-negative variant lacking aa 139–183, were kindly provided by Prof. Alan Hall (University College London, UK), and subcloned into the pCAG-Flag vector. RAC-binding regions (RBRs) in human *PAK1* (aa 67–150), human *MLK2* (aa 401–550), human *IRSp53* (aa 2–228), rat *N-WASP* (aa 191-270), mouse *RTKN* (Rhotekin) (aa 10–100) and human *ROCK* (Rho-associated coiled-coil containing protein kinase 1) (aa 67–150) were amplified by PCR from a cDNA pool of U251MG cells, rat brain or mouse brain, and constructed into the pGS21a vector (GenScript). For gene transcription analysis, pGL4.74[hRluc/TK] (control reporter plasmid), pGL4.44[luc2P/AP1-RE/Hygro] (AP1-luciferase reporter plasmid) and pGL4.32[luc2P/NF-κB-RE/Hygro] (NFkB-luciferase reporter plasmid) were purchased by Promega. The SRF reporter element, 5′-CAGACAGACGTGTTCTTAAGTCCATATTAGGACATCTACCATGTCCATATTAGGACATCTACTATGTCCATATTAGGACATCTTGTATGTCCATATTAGGACATCTAAAATGTCCATATTAGGAC-3′, was inserted into pGVB (Toyo, Inc) to yield SRF-luciferase reporter plasmid. All constructs were verified by DNA sequencing.

### Antibodies and histochemical reagents

The following antibodies were used: anti-GFP (Medical and Biological Laboratories Cat #598, RRID: AB_591819 or Nacalai Tesque, Cat #04404-84, RRID: AB_10013361), anti-NeuN (Millipore, Cat #MAB377, RRID:AB_2298772) and anti-Myc (Medical and Biological Laboratories, Cat #M047-3, RRID: AB_591112). Alexa Fluor 488 and 568 (Invitrogen) were used as secondary antibodies. 4′,6-diamidino-2-phenylindole (DAPI; Nichirei Bioscience) was used for staining DNA. Rhodamine-phalloidin (Invitrogen) was used for staining filamentous actin.

### GTP-hydrolysis and GTP/GDP-exchange assays

Preparation and purification of His-tag-fused RAC3 and the 11 variants were carried out according to the manufacturer’s instructions. To assess the basal GTP/GDP-exchange reactions, release of methylanthraniloyl (mant)-GDP (Sigma-Aldrich) was measured.^25^ Briefly, loading of RAC3 proteins with ^mant^GDP was performed by incubation for 90 min at 20°C in ^mant^GDP loading buffer [20 mM HEPES-NaOH (pH 7.5), 50 mM NaCl, 0.5 mM MgCl_2_, 5 mM EDTA, 1 mM dithiothreitol, 20-fold excess of ^mant^GDP] and subsequently adding 10 mM MgCl_2_ (final concentration) to stop the reaction. Remaining non-bound nucleotide was removed from the incubation mixture with a NAP-5 column (GE Healthcare Life Sciences). ^mant^GDP loaded GTPase was incubated with GppNHp (Abcam), which has 100 times higher concentration than that of RAC3 proteins, and fluorescence (365/450 nm) change was measured using an MTP-800 microplate reader (Corona). Intrinsic GTP-hydrolysis activity was then undertaken by directly measuring changes in the GTP concentration with a luciferase-coupled GTPase assay kit (GTPase-Glo Assay Kit, Promega) according to the manufacturer’s instructions.^26^ In short, 5 μM GTP and serially diluted RAC3 proteins were incubated in GTPase/GAP buffer for 60 min at room temperature. Once the GTP-hydrolysis reaction was completed, the remaining GTP was converted to ATP by adding GTPase-Glo Reagent. Subsequently, generated ATP was detected by the luciferase/luciferin-based reagent using a Turner Designs TD-20/20 luminometer (BMG Labtech).

### Cell culture and transfection

COS7 (monkey kidney fibroblast-like cell) and primary hippocampal neurons derived from embryonic day (E) 16.5 mice were cultured as described.^27^ Transient transfection was carried out using polyethyleneimine ‘MAX’ reagent (for COS7 cells) (Polysciences Inc) or Neon transfection system (for primary neurons) (Invitrogen).

### Pull-down assay of GTP-bound RAC3 and the variants

Glutathione S-transferase (GST)-fused RBRs of PAK1, MLK2, IRSp53, N-WASP, RTKN and ROCK were expressed in *Escherichia coli* BL21 (DE3) strain. Recombinant GST-proteins were purified according to the manufacturer’s instructions. COS7 cells were transfected with pCAG-Myc-RAC3, RAC3-G12R, -P29L, -P34R, -A59G, -G60D, -Q61L, -E62del, -E62K, -D63N, -Y64C or -K116N (1.0 μg/60 mm-dish). After 24 h, cells were lysed with the pull-down buffer (50 mM Tris-HCl, pH7.5, 150 mM NaCl, 5 mM MgCl_2_, 0.1% SDS, 1% Nonidet P-40 and 0.5% deoxycholate), and insoluble materials were removed by centrifugation. The supernatant was then incubated for 30 min at 4°C with Glutathione Sepharose 4B beads (GE Healthcare Life Sciences) with which GST-fused RBR of PAK1, MLK2, IRSp53, N-WASP, RTKN or ROCK was bound. The beads were washed twice with the pull-down buffer, and bound proteins were analysed by western blotting. Images were captured with an LAS-4000 luminescent image analyser (GE Healthcare Life Sciences).

### Assay of SRF, AP1 and NFkB-mediated gene transcription

Assays were performed as described.^28^ The control reporter vector was co-transfected into COS7 cells seeded on 24-well plates with the indicated RAC3 expression plasmid (0.1 μg/well) together with the SRF-, AP1- or NFkB-luciferase reporter plasmid. After transfection, cells were washed once with phosphate-buffered saline (PBS) and lysed with the passive lysis buffer according to the manufacturer’s instructions. Luciferase activities were determined with the dual-luciferase reporter assay system (Promega).

### *In utero* electroporation

The surgery on pregnant mice and embryo manipulation in the uterus were performed as previously described.^29,30^ At E14, pregnant ICR mice provided by Japan SLC were deeply anaesthetized with a mixture of three drugs: medetomidine (0.75 mg/kg), midazolam (4 mg/kg) and butorphanol (5 mg/kg).^31^*In utero* electroporation was then performed in the specific-pathogen-free animal facilities as described.^32^ Briefly, 1 µl of solution containing 0.1 μg of pCAG-Myc vector (control), pCAG-Myc-RAC3, -D63N, -E62del, -Y64C or -Q61L was injected with pCAG-EGFP (0.5 μg) into the lateral ventricle of mouse embryos with a glass micropipette made from a microcapillary tube (GD-1; Narishige). After the embryo in the uterus was placed between the tweezers-type disc electrode (5 mm in diameter) (CUY650-5; NEPA Gene), electronic pulses (50 ms of 35 V) were charged five times at 450 ms intervals with an electroporator (NEPA21; NEPA Gene). In this method, plasmids are introduced into the somatosensory area, which is included in the parietal lobe. Brains were fixed at indicated postnatal days, sectioned, and then analysed. As for the quantification of distribution of GFP-positive cells in brain slices, coronal sections of cerebral cortices containing the labelled cells were classified into three bins. The number of labelled cells in each region of at least three slices per brain was counted. A graphical timeline of the study design is shown (Supplementary Fig. 1). All experimental procedures were carried out in the daytime. Animals were neither excluded nor died during experimentation.

### Time-lapse imaging

After *in utero* electroporation at E14, organotypic coronal slices (250 µm-thickness) were prepared at E16 from the interventricular foramen with a microtome, placed on an insert membrane (pore size, 0.4 µm; Millipore), mounted in agarose gel, and cultured. The dishes were then mounted in an incubator chamber (5% CO_2_ and 40%O_2_, at 37°C) fitted onto an FV1000 confocal laser microscope (Olympus) and the primary somatosensory cortex was examined as described.^33^ Approximately 10–15 optical Z sections were acquired automatically every 15 min for 15 h, and about 10 focal planes (∼50 µm-thickness) were merged to visualize the entire shape of the cells.

### Quantitative analysis of axon growth

For estimation of axon growth, GFP signal intensity of the callosal axons was measured at P0 or P7 using ImageJ software in distinct regions (bin 1–3 or bin 1–4). The relative intensities of bins were normalized with bin 1 as 1.0 and compared using R software.

### Immunofluorescence

Immunofluorescence analysis was conducted essentially as described.^27^ Images of cultured cells were captured with a BZ-9000 microscope (Keyence) or an LSM-880 confocal laser microscope (Carl Zeiss). As for the cortical slice staining, brains were embedded in 3% agarose, cut into sections (100 μm-thickness) with a vibratome and photographed with an LSM-880 confocal laser microscope. Acquired images were analysed with ImageJ to determine cell morphological descriptor and fluorescence intensity.

### Statistical analysis

For all cell imaging experiments, cell counting and traces were assessed in a blinded manner by a technical staff who were not aware of experimental conditions. Statistical significance for multiple comparisons was determined by Dunnett’s or Tukey’s test (https://cran.r-project.org/web/packages/multcomp/multcomp.pdf). Comparisons between two groups were performed with Welch’s *t*-test.^34^*P* < 0.05 was considered statistically significant. Statistical analyses were performed using R (https://intro2r.com/citing-r.html; https://cran.r-project.org/doc/FAQ/R-FAQ.htmlCiting-R).

### Data availability

The data that support the findings of this study are available from the corresponding authors, upon reasonable request.

## Results

### Patient report

The study cohort consisted of 10 patients presenting with a syndromic NDD characterized by a moderate-to-severe psychomotor delay affecting the achievement of all the developmental milestones and resulting in global cognitive impairment (Supplementary Table 1).

**Figure 1:**
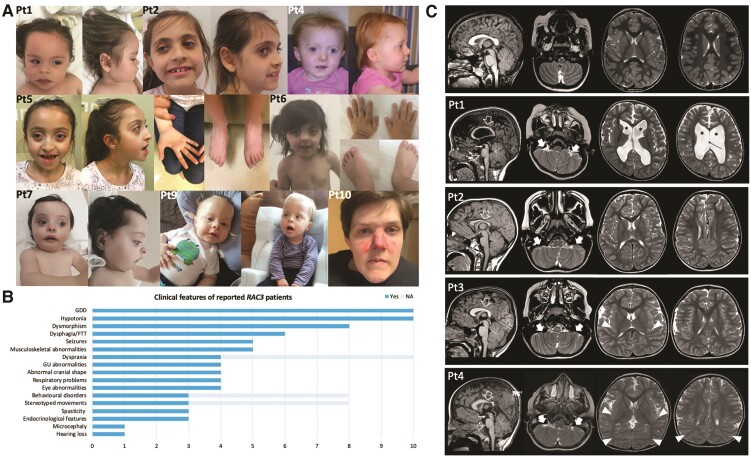
**Clinical and neuroimaging features of individuals with *RAC3*-related disorder.** (**A**) Dysmorphic features in subjects harbouring pathogenic *RAC3* variants. Patient 1 shows wide forehead with frontal bossing, upslanted palpebral fissures, depressed nasal bridge, anteverted nares, prominent philtrum and upper lip, and thick earlobes. Patient 2 is microcephalic and presents with palpebral ptosis, synophrys, deep-set eyes, prominent nasal tip with hypoplastic nasal wings, large and prominent ears and pointed chin. A round face with facial asymmetry, frontal bossing, high anterior hairline, upslanted palpebral fissures, depressed nasal bridge, micrognathia and posteriorly rotated ears can be observed in Patient 4. Patient 5 has facial hypotonia, frontal bossing, bilateral ptosis, proptosis, hypertelorism, shallow orbits, midface hypoplasia and pointed chin. Skeletal features consist of arachnodactyly and pes planus. Patient 6 has prominent eyes, long eyelashes, hypertelorism, midface hypoplasia, short nose, wide nasal bridge, anteverted nares, long philtrum, micrognathia and dental anomalies. She also has clinodactyly and foetal finger and toe pads. Patient 7 displays scaphocephaly with a wide forehead and a high anterior hairline, broad nasal base, anteverted nares, retrognathia and full lips. A prominent forehead with frontal bossing, epicanthal folds, deep philtrum, micrognathia with horizontal chin crease and posteriorly rotated ears are evident in Patient 9. Patient 10 shows down-slanted palpebral fissures, simplified ear helices, sharp tip of the nose, flat and long philtrum and pointed chin. (**B**) Graphic illustration of the most common clinical findings in the reported study participants: the bar graph shows the distribution of the cardinal clinical features, with the grey lines representing not available (NA) data. (**C**) Brain MRI, sagittal T_1_-weighted and axial T_2_-weighted images of Patients 1–4 and of a normal individual (*first row*) for comparison. The corpus callosum is shorter and thinner in all patients, especially at the level of the splenium (empty arrows), with thick genu in Patients 3 and 4. Note the absence of the anterior commissure in all patients. The volume of the cerebral white matter is reduced in all patients, with marked ventricular enlargement (asterisks) and septal defect (black thin arrow) in Patient 1. Diffuse dysgyria is present in all subjects, associated with polymicrogyria in the posterior regions in Patients 3 and 4 (arrowheads). Bilateral cerebellar folia abnormalities are noted in the inferior portions of the cerebellum in all patients, especially on the left side (thick arrows). There is hypoplasia of the midbrain in Patients 1 and 4 (white thin arrows) and a small pons in Patients 1, 2 and 4 (empty arrowheads). Mild enlargement of the subarachnoid spaces is noted in all patients, while brachycephaly is present only in Patients 3 and 4.

Facial dysmorphism was present in 8/10 individuals and recurrent features included wide forehead with frontal bossing and high anterior hairline, prominent eyes with upslanted palpebral fissures, arched eyebrows, long eyelashes, midface hypoplasia, broad nasal bridge and anteverted nares (Fig. 1A). Gross motor development was impaired in all individuals, who also showed a significant deficiency in expressive language, with three patients (Patients 1, 7 and 9) being nonverbal. A mild improvement of motor and verbal skills over time was common, although a regression limited to the phonological skills was observed in Patient 1. In Patients 4, 5 and 8, VABS administration revealed an overall low level of communication skills (scores range 34–68), daily living skills (scores range 27–56) and social skills and relationships (scores range 40–64) (Supplementary material). Behavioural abnormalities mainly consisted of ASD, aggressive/self-injurious behaviour and hypersensitivity. Feeding difficulties due to weak sucking or dysphagia were particularly relevant in the neonatal period, leading to failure to thrive in most individuals.

Neurological examination revealed hypotonia in all patients (Fig. 1B), frequently of neonatal onset, whereas appendicular spasticity was detected in only two patients (Patients 2 and 7). Additional neurological features included dyspraxia with poor visuospatial coordination (Patients 1–4), sleep disorders (Patients 5 and 7), irritability (Patient 6) and eyelid clonus (Patient 9). Stereotyped movements of the mouth and upper limbs were present in three patients (Patients 1, 2 and 9) (Supplementary Videos 1 and 2). Five out of 10 individuals (Patients 1, 5, 6, 9 and 10) experienced recurrent seizures. Age at onset ranged from the first day of life to 10 months and semiology was variable, including infantile spasms, clonic, myoclonic and tonic seizures. EEG showed focal/multifocal epileptic discharges, sometimes with secondary generalization. Hypsarrhythmia or slowed background were also observed in the most severely affected individuals (Patients 1 and 5). In all individuals, seizures were responsive to antiseizure medications, e.g. valproate, vigabatrin, phenobarbital or levetiracetam.

Progressive microcephaly was detected in one individual (Patient 2), with others having abnormal cranial shape in the form of plagiocephaly (Patients 1 and 4), brachycephaly (Patient 5) and scaphocephaly (Patient 7). Additional syndromic features were also evident in most individuals. Among the musculoskeletal abnormalities, scoliosis (Patients 1, 3 and 10), vertebral defects (Patient 1), pes planus (Patients 3 and 5) and joint laxity (Patients 3 and 4) were recurrent. Ocular involvement consisted of horizontal nystagmus (Patients 1 and 5), strabismus (Patients 1, 3 and 4) and astigmatism with hypermetropia (Patients 1 and 3). Some individuals presented with genitourinary abnormalities, such as genital hypoplasia (Patient 6), follicular cysts (Patient 5) and enuresis (Patients 3 and 4). Endocrinological features were less common and encompassed truncal obesity (Patient 4), precocious puberty (Patient 5) and hyperthyroidism with advanced bone age (Patient 10). Respiratory involvement was observed in four patients, who experienced recurrent respiratory tract infections (Patients 3 and 7) and apnoeic episodes (Patients 9 and 10).

Although clinical features of *RAC3*-related disorder have been well documented in infancy, limited follow-up information is available and little is known about the natural history of the condition. Herein we report the oldest known affected individual (Patient 10, aged 29 years), who seems to confirm three aspects of NEDBAF. First, the psychomotor delay may mildly improve over time, whereas regression is unusual (only observed in Patient 1 from our cohort, and limited to phonological skills). Second, seizures are responsive to antiepileptic treatment and have a generally benign course. Third, premature death does not appear to be part of the core clinical spectrum, although the apnoeic episodes reported in a minority of subjects are potentially life-threatening. Additional studies reporting large case series will determine if these observations are valid.

As summarized in Supplementary Tables 2 and 4, affected individuals present with hypotonia, weak cry or poor sucking in the neonatal period. Feeding difficulties are common and may fail to thrive (62.5%). During the first year of life, global developmental delay with a severe involvement of language skills becomes apparent, but psychomotor arrest or regression are not typical. Neurological examination shows hypotonia (93.7%), dyspraxia (40%), or spasticity (25%) (Supplementary Fig. 2). Seizures occur in 43.7% of individuals, as early as the newborn period in two patients. Behavioural disorders and stereotyped movements are seen (both in 27.3% of subjects). Dysmorphic features are prominent (81.3%) and suggestive of a peculiar facial gestalt. Although microcephaly is rare (12.5%), an abnormal cranial shape is observed in 31.2% of individuals, but rarely reflects true craniosynostosis. Associated syndromic features are particularly relevant in some patients and include musculoskeletal (43.7%), genitourinary (37.5%), ocular (25%) and endocrinological (18.7%) abnormalities. Of note, respiratory problems are present in 31.2% of individuals. The clinical features of 16 individuals reported in this and previous studies were summarized in Supplementary Table 2.

### Neuroimaging

Callosal anomalies were present in 10/10 participants, including hypoplasia with prevalent posterior involvement (eight cases), thick and short morphology (one case, Patient 3) and partial agenesis (one case, Patient 10) (Fig. 1C). White matter thinning and enlarged subarachnoid spaces with global reduction of the brain volume were noted in 8/10 and 9/10 cases, respectively. Malformations of cortical development were noted in 7/10 participants, including polymicrogyria involving the posterior regions (five cases), diffuse dysgyria (four cases), and small grey matter heterotopias dispersed in the cerebral white matter (one case, Patient 1). Cerebellar dysplasia with foliation abnormalities involving the inferior cerebellar hemispheres was detected in 6/10 participants, while brainstem anomalies characterized by a small midbrain and/or pons were seen in 5/10 cases. Chiari I anomaly was not detected in this series, but mild (<5 mm) caudal descent of the cerebellar tonsils was noted in three participants. Neuroimaging features of 16 cases reported in this and previous studies were summarized in Supplementary Table 2.

### Identification and analyses of *RAC3* variants

Based on allele frequency (<0.001 in gnomAD), involvement of conserved amino acid residues, and predicted impact on protein function, variants in *RAC3* were the most plausible candidate variants in the study cohort. NGS led to the identification of seven distinct variants, including the previously reported c.184G>Ap.E62K in Patient 7 (ClinVar ID: 425149)^18^. Most of the novel variants were missense: c.187G>A p.D63N (Patients 1 and 9); c.191A>Gp.Y64C (Patient 3); c.179G>Ap.G60D (Patient 4); c.34G>Cp.G12R (Patient 5); c.348G>Cp.K116N (Patient 8) (Supplementary Fig. 3). All these variants are rare, affect conserved residues (GERP scores 2.5–3.91) (Supplementary Fig. 4), and are predicted to have a high pathogenic impact (CADD score 24.3–27.6) (Supplementary Table 3). The missense variant c.176C>Gp.A59G previously detected in Patient 6^20^ is also predicted to be pathogenic by several *in silico* tools (Supplementary Table 3). In Patient 2, the novel in-frame deletion c.186_188delGGAp.E62del was detected. This variant results in a protein coding length change through the deletion of the conserved Glu62 (GERP score 3.79). No copy number variants with a pathogenic potential could be detected in the reported subjects (Supplementary Table 1 and Supplementary material).

A common structural and biochemical feature across the small GTPase superfamily is the G domain, which is defined by five G boxes with certain structurally conserved amino acid residues (Supplementary Fig. 3). The G1 box/P-loop (GxxxxGKS/T, where x is any amino acid) recognizes the β-phosphate and Mg^2+^ ion of guanine nucleotides. The G2 box (T) positioned in the Switch I region interacts with the γ-phosphate and Mg^2+^, while the G3 box (DxxGQ/H/T) localizing in the Switch II region is responsible for GTP-hydrolysis. Eventually, the G4 (T/NKxD) and G5 (C/SAK/L/T) boxes make specific contacts with the guanine base. The previously reported p.P34R and p.P29L variants^18,19^ lead to substitutions of evolutionarily conserved amino acids in the Switch I region, with p.P34R being also included in the effector binding region. It is of note that all variations identified in this study occurred at residues highly conserved across species (Supplementary Figs 3 and 4). Among the *RAC3* variants observed in our cohort, seven variants (p.A59G, p.G60D, p.Q61L, p.E62del, p.E62K, p.D63N and p.Y64C) are mapped to the Switch II region, which is recognized as a variation hot spot among *RAC1*, *RAC3* and *CDC42*.^16,35,36^ In addition, the p.G12R and p.K116N are the first variants affecting the conserved residues in the G1 and G4 boxes, respectively (Supplementary Figs 3 and 4).

### *In vitro* biological and biochemical activities of the 11 disorder causative *RAC3* variants

To get further insights into the pathophysiological significance of *RAC3* variants associated with NDDs, activation states were first examined *in vitro* for the eight variants identified in the enrolled patients, together with the recently reported p.P34R^19^, p.P29L and p.Q61L^18^ variants. Of note, the p.Q61L variant is best characterized as a GTPase-defective constitutively active version of RAC1 involved in tumour cell metastasis and invasion.^37–39^

**Figure 2:**
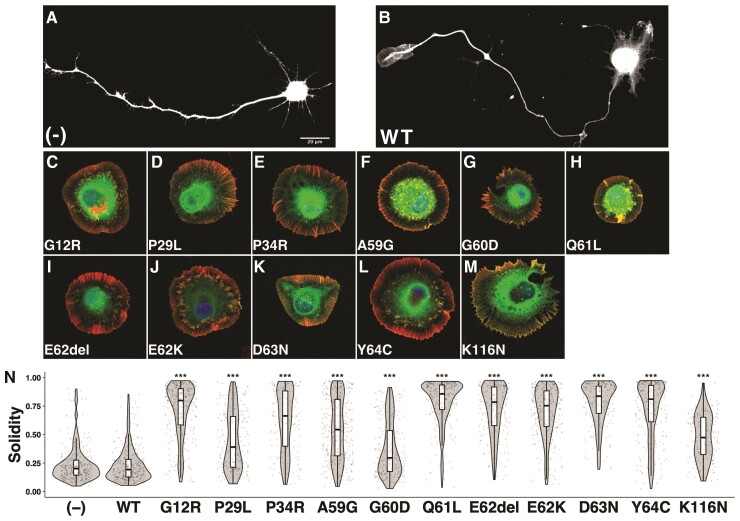
**Effects of the disease-causative 11 RAC3 variants on neuronal morphology *in vitro.*** (**A**–**M**) Dissociated hippocampal neurons from E16 mice were co-electroporated with pCAG-EGFP (0.1 μg) together with pCAG-Myc (−), pCAG-Myc-RAC3 (wild-type, WT), -G12R, -P29L, -P34R, -A59G, -G60D, -Q61L, -E62del, -E62K, -D63N, -Y64C and -K116N (0.3 μg each). Cells were fixed at 3 days *in vitro* (DIV), and co-stained with anti-GFP (green), rhodamine-phalloidin (red) and DAPI (blue). Scale bar = 20 μm. (**N**) Quantification of (**A**–**M**). The morphological descriptor (solidity) of GFP-positive neurons (≥200 cells each) was shown in violin plot with boxplot. ‘Solidity’ is the ratio of the area of a cell to the area of a convex hull of the cell. Values of the ratio were calculated by ‘Analyze Particles…’ method in ImageJ (https://imagej.nih.gov/ij/docs/menus/analyze.html). The significance of difference between (−) and each variant was determined using Dunnett’s test. WT versus (−), *P* = 1; G12R versus (−), *P* < 1 × 10^−10^; P29L versus (−), *P* < 1 × 10^−10^; P34R versus (−), *P* < 1 × 10^−10^; A59G versus (−), *P* < 1 × 10^−10^; G60D versus (−), *P* < 1 × 10^−10^; Q61L versus (−), *P* < 1 × 10^−10^; E62del versus (−), *P* < 1 × 10^−10^; E62K versus (−), *P* < 1 × 10^−10^; D63N versus (−), *P* < 1 × 10^−10^; Y64C versus (−), *P* < 1 × 10^−10^; K116N versus (−), *P* < 1 × 10^−10^. ***P < 0.001.

When control pCAG-Myc vector was expressed in primary cultured hippocampal neurons, cells were normally differentiated in a time-dependent manner (Fig. 2A and N). Likewise, neurons expressing wild type RAC3 were normally differentiated with single axon and numerous dendrites, although its expression induced lamellipodia at the growth cone of axon and cell body (Fig. 2B). These results indicate that the basal activity of exogeneous wild type RAC3 had little effect on cell differentiation *in vitro*. In contrast, neurons transfected with the 11 studied variants displayed cell rounding with typical lamellipodia formation and less neurite extension, resulting in a prominent increase in the solidity (Fig. 2C–N). While the median of solidity was shifted upward when each variant was expressed, the degree was variant-dependent because cells expressing some variants such as RAC3-P29L, -A59G, -G60D and -K116N occasionally showed neurite extension (Supplementary Fig. 5). Taken together, all the 11 variants appeared to facilitate cytoskeletal reorganization to form lamellipodia to greater or lesser degrees. The respective variants should dysregulate intracellular signalling in variant-dependent manners and adversely affect neuronal morphology and function.

**Figure 3:**
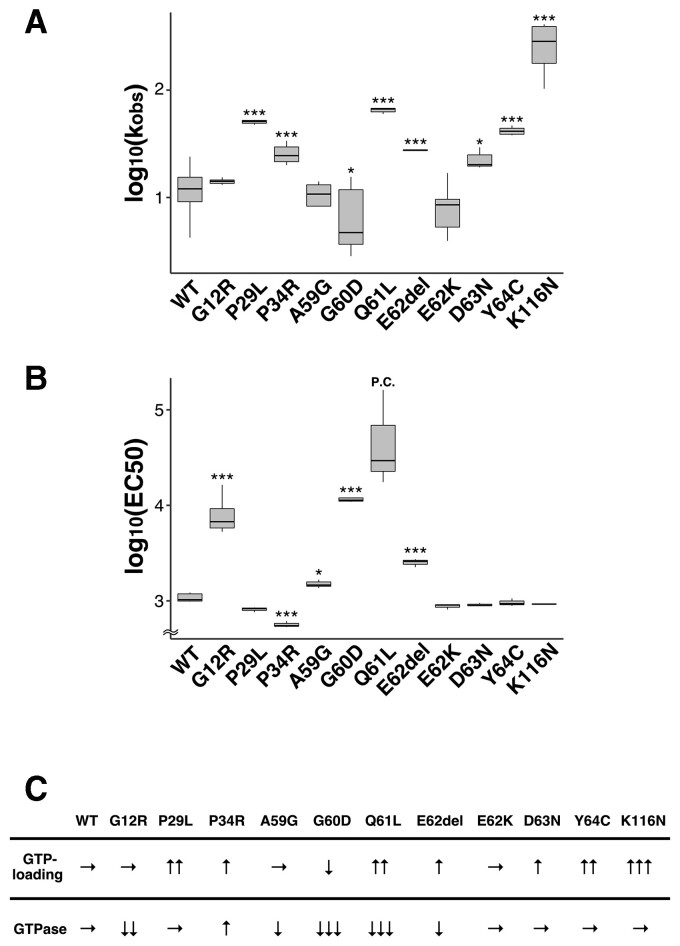
**Characterization of activation states of the disease-causative 11 RAC3 variants *in vitro.*** (**A**) Measurement of GDP/GDP-exchange activity. Recombinant His-tag-fused RAC3 (wild-type, WT) and the 11 variants (RAC3-G12R, -P29L, -P34R, -A59G, -G60D, -Q61L, -E62del, -E62K, -D63N, -Y64C and -K116N) were preloaded with fluorescent ^mant^GDP and incubated with unlabelled GTP. Intrinsic nucleotide exchange was measured and ^mant^GDP-dissociation rates of wild-type and respective variants were calculated as observed rate constants [k_obs_ (×10^−5^ s^−1^)] from the results in Supplementary Fig. 5A. Number of replicates, *n* ≥ 4. The significance of difference between wild-type and each variant was determined using Dunnett’s test. G12R versus WT, *P* = 0.8305; P29L versus WT, *P* < 0.001; P34R versus WT, *P* < 0.001; A59G versus WT, *P* = 0.7655; G60D versus WT, *P* = 0.0135; Q61L versus WT, *P* < 0.001; E62del versus WT, *P* < 0.001; E62K versus WT, *P* = 0.1018; D63N versus WT, *P* = 0.0132; Y64C versus WT, *P* < 0.001; K116N versus WT, *P* < 0.001. *P < 0.05, ***P < 0.001. (**B**) Measurement of GTP-hydrolysis activity. Intrinsic GTP-hydrolysis activity was analysed by direct measuring of changes in the GTP concentration with GTPase-Glo assay kit. EC50 (half maximal effective concentration) was estimated from the sigmoidal fitting curve in Supplementary Fig. 5B. Number of replicates, *n* ≥ 3. *P*-value was calculated as in (**A**). G12R versus WT, *P* < 1 × 10^−04^; P29L versus WT, *P* = 0.174; P34R versus WT, *P* < 1 × 10^−04^; A59G versus WT, *P* = 0.012; G60D versus WT, *P* < 1 × 10^−04^; E62del versus WT, *P* < 1 × 10^−04^; E62K versus WT, *P* = 0.514; D63N versus WT, *P* = 0.743; Y64C versus WT, *P* = 0.952; K116N versus WT, *P* = 0.833. *P < 0.05, ***P < 0.001. P.C. = positive control. (**C**) Summary of the results in **A** and **B**. → = no change; ↑ = activity increased; ↓ = activity decreased.

We then performed biochemical analyses and measured GTP/GDP-exchange and GTP-hydrolysis activity. When the effects of these 11 variations on GTP/GDP-exchange activity were examined with recombinant RAC3 proteins, the assay documented a variably increased or decreased activity. When compared with wild type RAC3, log_10_(k_obs_: observed rate constant) was especially high for RAC3-K116N and moderately high for RAC3-Q61L, -P29L, -Y64C, -E62del, -P34R and -D63N in this order, indicating an accelerated exchange reaction (Fig. 3A and Supplementary Fig. 6A). In contrast, a statistically significant decrease of log_10_(k_obs_) was observed for RAC3-G60D, indicating suppression of the exchange reaction (Fig. 3A and Supplementary Fig. 6A). The exchange activity of RAC3-G12R, -A59G and -E62K did not show statistical difference in comparison to the wild type (Fig. 3A and Supplementary Fig. 6A). Then, GTP-hydrolysis activity was assayed for each variant. This was highly suppressed by p.Q61L, p.G60D and p.G12R, while moderate to weak suppression was observed for p.E62del and p.A59G (Fig. 3B and Supplementary Fig. 6B). We consider that p.G12R, p.G60D and p.Q61L primarily activated the protein by eliminating the GTP-hydrolysis activity like oncogenic RAS proteins. In contrast, the activity of RAC3-P34R was higher than the wild-type (Fig. 3B and Supplementary Fig. 6B). Of note, RAC3-P29L, E62K, -D63N, -Y64C and -K116N did not affect GTP-hydrolysis activity when compared to the wild-type (Fig. 3B and Supplementary Fig. 6B).

Based on the obtained results summarized in Fig. 3C, it is possible to categorize the biochemical properties of the 11 variants into three groups based on GTP/GDP-exchange activity. Group I encompasses RAC3-P29L, -P34R, -Q61L, -E62del, -D63N, -Y64C and -K116N. These variants demonstrated a higher GTP/GDP-exchange activity. While GTP-hydrolysis activity of RAC3-P29L, -D63N, -Y64C and -K116N was comparable to that of wild-type, RAC3-Q61L and -E62del showed lower hydrolysis activity. We assume that these seven variants are prone to be in GTP-bound active conformation, since they exhibited high GTP/GDP-exchange activity with GTP-hydrolysis activity equivalent to or lower than that of wild-type RAC3. On the other hand, although RAC3-P34R has a statistically high GTP-hydrolysis activity, we assume that the variant is activated due to the accelerated GTP/GDP exchange activity in the context of an intracellular environment in which the GTP concentration is substantially higher than that of GDP. It is notable that the substitution of the corresponding Pro34 in RAC2 with His was suggested to activate the protein based on crystal structure modeling.^40^ Group II includes RAC3-G12R, -A59G and -E62K, all showing a GTP/GDP-exchange activity similar to the wild-type. Since RAC3-G12R and -A59G displayed suppressed GTP-hydrolysis activity, they were likely to prefer GTP-binding. As for RAC3-E62K with normal GTP-hydrolysis and GTP/GDP-exchange activities *in vitro*, it appears that abnormal interaction with unidentified GEFs and/or GAPs holds RAC3-E62K in an active state in primary neurons. In this context, RAC2-E62K has been reported to be hyper-activated through altered GEF specificity and impaired GAP function.^41^ Eventually, Group III includes RAC3-G60D, which may prefer GTP-binding because of its very low GTP-hydrolysis activity even if the GTP/GDP-exchange rate is also low. Based on the results in Figs 2 and 3, we concluded that all the 11 variations confer a gain-of-function phenotype with respect to the RAC3 function. Biological and biochemical diversity among variations may variously dysregulate RAC3 function *in vivo*, leading to variation-specific clinical phenotypes.

### Possible interaction of the RAC3 variants with various downstream effectors

**Figure 4:**
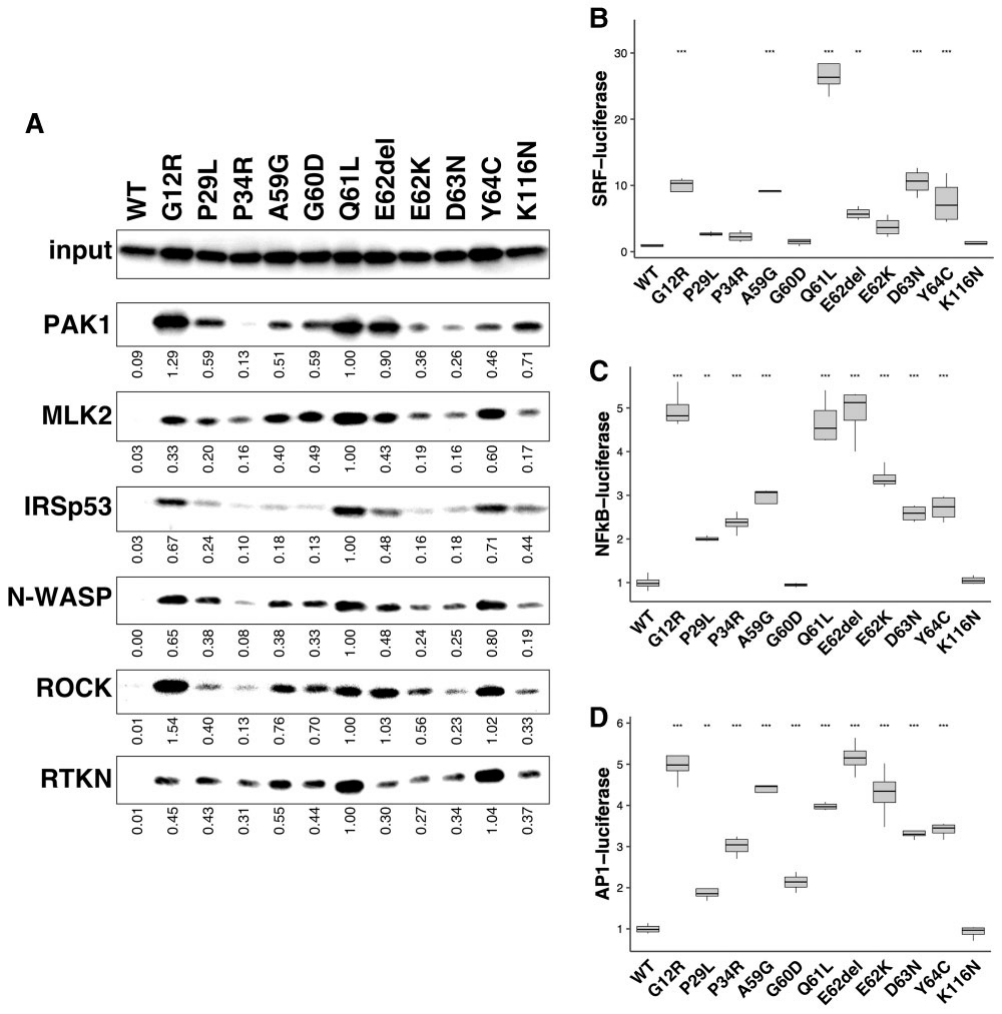
**Possible interaction of the disease-causative 11 RAC3 variants with various downstream effectors.** (**A**) Interaction with the RBRs of PAK1, MLK2, IRSp53, N-WASP, ROCK and RTKN. COS7 cells were transfected with pCAG-Myc-RAC3 (wild-type, WT), -G12R, -P29L, -P34R, -A59G, -G60D, -Q61L, -E62del, -E62K, -D63N, -Y64C and -K116N (0.3 μg each). Cell lysates were prepared, and the pull-down assay was conducted as described in the ‘Materials and Methods’ section with respective GST-fused RBRs (5 μg each). Bound RAC3 proteins were detected by western blotting with anti-Myc. RAC3 activity was indicated by the amounts of RAC3 bound to respective GST-RBRs, and total cell lysates were also immunoblotted with anti-Myc for normalization (*input*). Relative band intensity was shown when the value of RAC3-Q61L was taken as 1.0. (**B**–**D**) Involvement of the *RAC3* variations in SRF- (**B**), NFkB- (**C**) or AP1-dependent (**D**) gene transcription. COS7 cells were co-transfected with each luciferase expression vector together with pCAG-Myc-RAC3 (WT) or the expression vector for the 11 variants. Luciferase activity obtained with wild-type was taken as 1.0, and relative activities are shown as box plot. Number of replicates, *n* = 4. The significance of difference between wild-type and each variant was determined using Dunnet’s test. (**B**) G12R versus WT, *P* < 0.001; P29L versus WT, *P* = 0.81326; P34R versus WT, *P* = 0.94861; A59G versus WT, *P* < 0.001; G60D versus WT, *P* = 0.99999; Q61L versus WT, *P* < 0.001; E62del versus WT, *P* = 0.00997; E62K versus WT, *P* = 0.27663; D63N versus WT, *P* < 0.001; Y64C versus WT, *P* < 0.001; K116N versus WT, *P* = 0.99999. (**C**) G12R versus WT, *P* < 1 × 10^−04^; P29L versus WT, *P* = 0.00159; P34R versus WT, *P* < 1 × 10^−04^; A59G versus WT, *P* < 1 × 10^−04^; G60D versus WT, *P* = 1.00000; Q61L versus WT, *P* < 1 × 10^−04^; E62del versus WT, *P* < 1 × 10^−04^; E62K versus WT, *P* < 1 × 10^−04^; D63N versus WT, *P* < 1 × 10^−04^; Y64C versus WT, *P* < 1 × 10^−04^; K116N versus WT, *P* = 1.00000. (**D**) G12R versus WT, *P* < 1 × 10^−04^; P29L versus WT, *P* = 0.00275; P34R versus WT, *P* < 1 × 10^−04^; A59G versus WT, *P* < 1 × 10^−04^; G60D versus WT, *P* = 0.00017; Q61L versus WT, *P* < 1 × 10^−04^; E62del versus WT, *P* < 1 × 10^−04^; E62K versus WT, *P* < 1 × 10^−04^; D63N versus WT, *P* < 1 × 10^−04^; Y64C versus WT, *P* < 1 × 10^−04^; K116N versus WT, *P* = 0.99999. **P < 0.01, ***P < 0.001.

While the patients described in this study share core NDD phenotypes associated with callosal and cortical malformations, clinical features peculiar to each individual may be attributable to the position and type of the involved amino acids changes. Since the 11 *RAC3* variants analysed in this study are considered to be differently activated based on cell biological and biochemical analyses (Figs 2 and 3), we looked into the downstream signalling pathways interacting with these variants. To this end, a pull-down assay was performed to examine the interaction of respective RAC3 variants with recombinant RBRs of various effectors including PAK1, MLK2, IRSp53, N-WASP, ROCK and RTKN. Consequently, the *RAC3* variants were observed to interact with these RBRs in variant-dependent manners (Fig. 4A). The RBR of PAK1, which is crucial for neuronal migration and variated in patients with NDDs,^42–46^ was associated with all the variants except RAC3-P34R. High affinity was displayed for RAC3-G12R, -Q61L, -E62del and -K116N in this order, and low affinity was observed for RAC3-D63N (Fig. 4A). The RBR of MLK2, which activates the JNK-MAP kinase pathway downstream of RAC,^47^ significantly interacted with RAC3-Q61L and then -Y64C, and moderate to weak affinity was observed for all other variants (Fig. 4A). The RBR of IRSp53, an adaptor protein acting at the membrane-actin interface and related to the formation of filopodia and lamellipodia,^48,49^ showed relatively high affinity towards RAC3-Q61L, -Y64C and -G12R (Fig. 4A). Although RAC3-E62del and -K116N had moderate affinity, a very weak interaction was observed with other variants (Fig. 4A). The RBR of N-WASP, a regulator of actin polymer reorganization in the cytoplasm and nucleus,^50,51^ displayed relatively strong affinity to RAC3-Q61L, -Y64C and -G12R in this order, while weak to moderate affinity was detected for all other variants but -P34R (Fig. 4A). The RBR of ROCK, a key regulator of actin cytoskeleton and cell polarity,^52,53^ exerted strong binding to RAC3-G12R, -Q61L, -E62del and -Y64C (Fig. 4A). While moderate interaction was shown for RAC3-A59G, -G60D and -E62K, other variants except RAC3-P34R showed a weak affinity (Fig. 4A). RTKN has been shown to regulate the septin cytoskeleton.^54^ Although RTKN was first reported as a Rho-specific effector,^55^ we have found that RTKN interacts with activated RAC3 but not RAC1 through the Rho-binding domain. We thus asked whether this domain, which is referred to as RBR here, interacts with the 11 variants. We determined that RTKN-RBR interacted with the 11 variants, with particularly strong affinity to RAC3-Q61L and -Y64C (Fig. 4A). Taken together, given that the respective *RAC3* variants are activated versions, they are strongly suggested to hyper-activate different sets of effectors in variant-dependent manners and, thereby, dysregulate specific downstream signalling pathways in context-dependent manners. Full cropped data of western blotting were depicted in Supplementary Fig. 7.

We next examined signal transduction pathways underlying the aberrant activation states caused by these variations. We focused on SRF-, NFkB- and AP1-mediated gene expression, since their related signalling pathways include Rho-family proteins and MAP kinases.^56–58^ When effects of respective variants on SRF-dependent gene transcription were assessed, prominent transcriptional activation was observed in cells expressing RAC3-Q61L, while moderate activation was induced by RAC3-G12R, -A59G, -E62del, -D63N and -Y64C (Fig. 4B). In contrast, RAC3-P29L, -P34L, -G60D and -K116N had marginal effects. Meanwhile, NFkB-dependent gene transcription was highly increased by RAC3-E62del, -G12R and -Q61L (Fig. 4C). Noteworthy, these three variants were observed to show high affinity to PAK1-RBR (Fig. 4A). Other variants except for RAC3-G60D and -K116N, which showed no effects, demonstrated moderate NFkB-activation (Fig. 4C). Then, when we looked into the effects on AP1-mediated gene expression, each *RAC3* variant induced the expression similar to that of NFkB; the *RAC3* variants other than RAC3-A59G, -G60D, -Q61L and -E62K might share common signalling pathways in terms of NFkB and AP-1 (Fig. 4C and D). These results further support the hypothesis that the disease-causing *RAC3* variants may drive different and/or common downstream signalling pathways, leading to variant-dependent dysregulation of cellular processes and, eventually, common as well as patient-specific clinical phenotypes.

### *In vivo* effects of the four *RAC3* variants in the Switch II region on neuronal migration and morphology during corticogenesis

**Figure 5:**
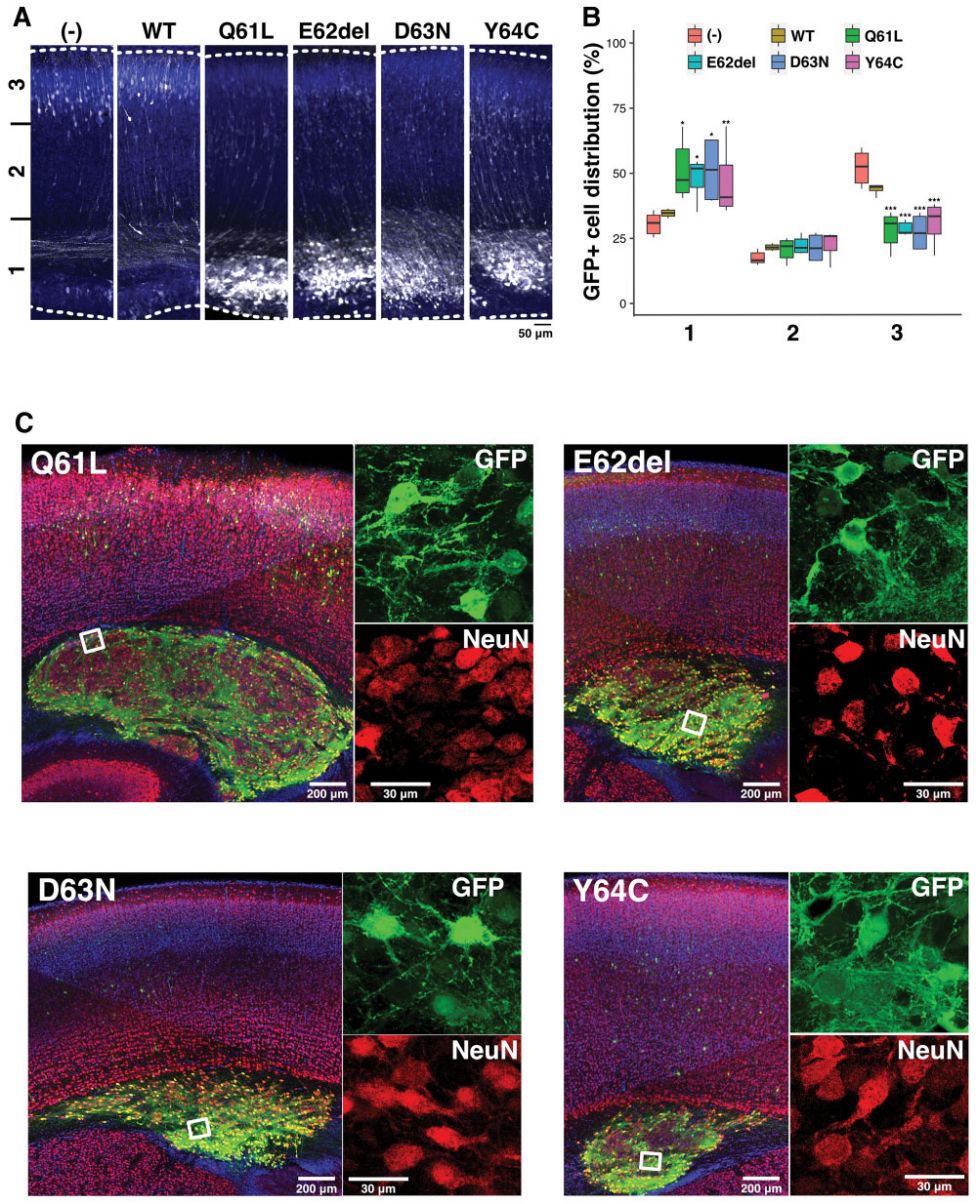
**Effects of the four RAC3 variants in the Switch II region on excitatory neuron migration during corticogenesis.** (**A** and **C**) Migration defects of neurons expressing each variant. pCAG-EGFP (0.5 μg) was co-electroporated *in utero* with pCAG-Myc (−), pCAG-Myc-RAC3 (wild-type, WT), -RAC3-Q61L, -E62del, -D63N or -Y64C (0.1 μg each) into the ventricular zone progenitor cells at E14.5. Coronal sections were prepared at P0 (**A**) or P7 (**C**). Coronal slices were double-stained with anti-GFP (white) and DAPI (blue) for **A** or triple-stained with anti-NeuN (red), anti-GFP (green) and DAPI (blue) for **C**. Boxed areas in the *left* panels were magnified in *right* panels (**C**). Scale bars = 50 μm (**A**), 200 μm (**C**, *left*) and 30 μm (**C**, *right*). **(B)** Quantification of the distribution of GFP-positive neurons in distinct regions of the cerebral cortex (bin 1–3) for each condition in **A**. Number of replicates, *n* ≥ 5. The significance of difference between control (−) and each variant was determined using Dunnett’s test and shown in box plot. (Bin 1) WT versus (−), *P* = 0.9498; Q61L versus (−), *P* = 0.0045; E62del versus (−), *P* = 0.0172; D63N versus (−), *P* = 0.0119; Y64C versus (−), *P* = 0.0356. (Bin 2) WT versus (−), *P* = 0.413; Q61L versus (−), *P* = 0.535; E62del versus (−), *P* = 0.185; D63N versus (−), *P* = 0.439; Y64C versus (−), *P* = 0.133. (Bin 3) WT versus (−), *P* = 0.212; Q61L versus (−), *P* < 0.001; E62del versus (−), *P* < 0.001; D63N versus (−), *P* < 0.001; Y64C versus (−), *P* < 0.001. ***P < 0.001, **P < 0.01, *P < 0.05.

Based on the data obtained from *in vitro* analyses, the disease-causative *RAC3* variants are most likely to be activated and induce abnormal neuronal morphology. Since cell morphology is closely associated with migration, we examined the effects of the *RAC3* variants on the migration of newly generated excitatory cortical neurons *in vivo*. We especially focused on the Switch II region and selected the p.Q61L, p.E62del, p.D63N and p.Y64C, since the region is a variation hotspot among *RAC1*, *RAC3* and *CDC42*. Using an *in utero* electroporation-mediated acute gene transfer method, pCAG-Myc-RAC3 or pCAG-Myc vector harbouring each variant was co-electroporated with pCAG-EGFP into the progenitor cells in the ventricular zone of E14.5 embryonic brains, and localization of transfected cells and their progeny was observed at P0. Neurons expressing the control vector or pCAG-Myc-RAC3 migrated normally to the superficial layer (bin 3; layers II/III) of the cortical plate (Fig. 5A). In contrast, most cells transfected with pCAG-Myc-RAC3-Q61L, -E62del, -D63N or -Y64C remained in the ventricular and subventricular zones and the intermediate zone (bin 1) (Fig. 5A). Quantitative analyses confirmed that each variant exhibited statistically similar effects (Fig. 5B). The result that the expression of the wild type did not statistically affect neuronal cell positioning indicates that the basal activity of RAC3 had no effects on neuronal cell migration and that physiological balance between GTP- and GDP-bound states of RAC3 should be essential for the establishment of cortical architecture during corticogenesis. Based on western blotting analyses with cortical neurons where Rac3 proteins were electroporated, the expression level of each protein was found to be comparable and lower than endogenous Rac3 (Supplementary Fig. 8).

Although cells expressing respective variants were dominantly distributed at bin 1, a small portion of such neurons still reached the superficial layer of cortical plate (Fig. 5A and B), perhaps due to a relatively low amount of the expression vector incorporated. Given that transfection efficiency into each cell depends on the size of the cell surface area which is physically exposed to the ventricular lumen (CSF) where plasmids are injected, neurons incorporating low amount of the expression vector are supposed to undergo partial effects of the variant.

When long-term effects of expression of the four RAC3 variants were examined, we noticed the formation of neuronal cell clusters in the ventricular and subventricular zones in developing cerebral cortex at P7 (Fig. 5C). The cells incorporated in the cluster were positive for NeuN, indicating they were differentiated at abnormal positions. These cells also extended neurites in the cluster (Fig. 5C). The results obtained indicate that the four variants prevent, rather than delay, cortical neuron migration, and that RAC3 plays a pivotal role in neuronal migration.

### Time-lapse imaging of migration of cortical neurons expressing the four *RAC3* variants in the Switch II region

**Figure 6:**
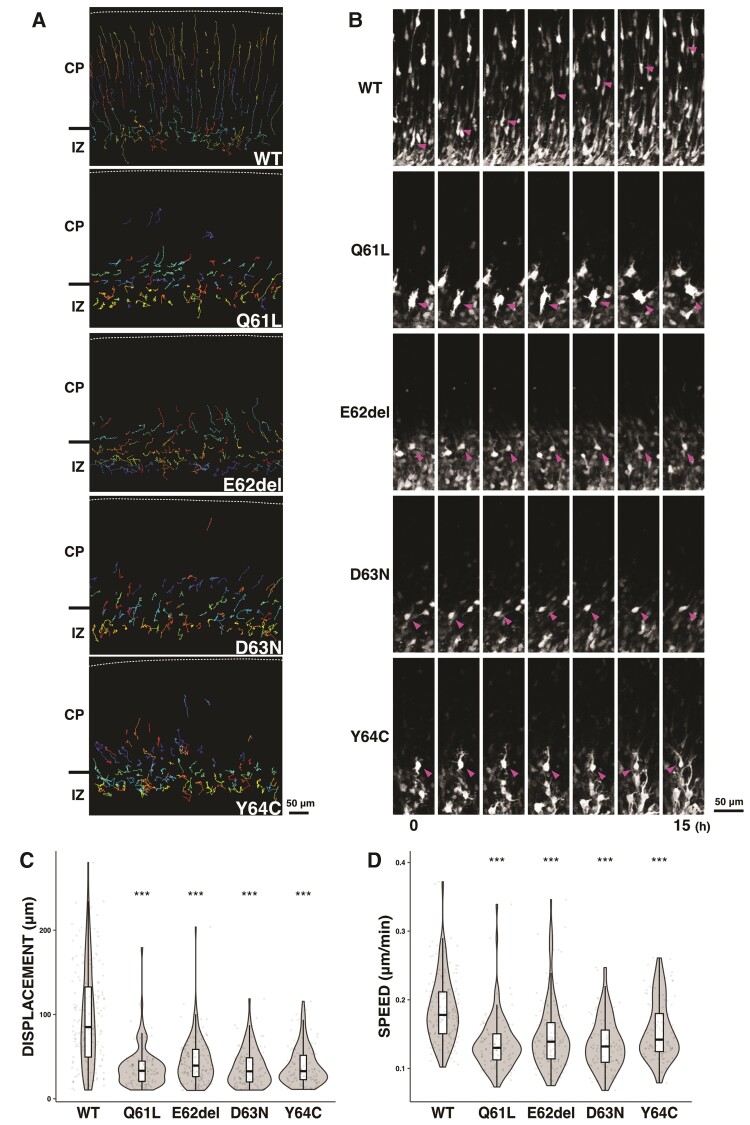
**Time-lapse imaging analyses of migration of cortical neurons expressing the four disease-causative RAC3 variants in the Switch II region.** Electroporation was performed as in Fig. 5. Analyses were repeated three times for each case and representative results were shown in (**A** and **B**). (**A**) Tracing of neurons expressing Myc-RAC3 (wild-type, WT), -RAC3-Q61L, -E62del, -D63N or -Y64C in upper intermediate zone (IZ)–lower cortical plate (CP). Migratory tracks of over 100 cells were demonstrated as colour lines. (**B**) Time-lapse imaging of neurons, which express Myc-RAC3 (WT) or the variants, migrating in the IZ–CP boundary. (**C**) Migration distance of neurons expressing Myc-RAC3 or the four variants. Over 100 cells selected in (**A**) were analysed. The distance was shown in violin plot with box plot. The significance of difference between wild-type and each variant was determined using Dunnett’s test. Q61L versus WT, *P* < 2 × 10^−16^; E62del versus WT, *P* < 2 × 10^−16^; D63N versus WT, *P* < 2 × 10^−16^; Y64C versus WT, *P* < 2 × 10^−16^. ***P < 0.001. (**D**) Migration velocity of cells expressing Myc-RAC3 or the four variants. Over 100 cells selected in (**A**) were analysed. The velocity was shown in violin plot with box plot. The significance of difference between wild-type and each variant was determined using Dunnett’s test. Q61L versus WT, *P* < 1 × 10^−07^; E62del versus WT, *P* < 1 × 10^−07^; D63N versus WT, *P* < 1 × 10^−07^; Y64C versus WT, *P* = 1.16 × 10^−07^. ***P < 0.001.

Newborn cortical neurons generated at the ventricular zone primarily exhibit multipolar shapes in the lower intermediate zone, where cells show a slow and irregular movement termed multipolar movement for ∼24 h.^59^ Neurons then transform into a bipolar shape with a leading process and an axon in the upper intermediate zone, move into the cortical plate, and exhibit a saltatory movement termed radial migration toward pial surface. Given the tight correlation between cell movement and morphology, the abnormal accumulation and cluster formation of the *RAC3* variant-expressing neurons in the subventricular zone and intermediate zone should be associated with impaired shape change into the bipolar status (multipolar-bipolar transition). We thus carried out time-lapse imaging to further investigate the morphology of cells stuck in the subventricular zone and intermediate zone during corticogenesis. To this end, ventricular zone progenitor cells were co-electroporated with pCAG-EGFP together with wild-type RAC3- or respective variant-expression vectors at E14.5, and cell migration was monitored from E16 for 15 h in the subventricular zone, intermediate zone and lower cortical plate. Consequently, time-lapse imaging revealed clear differences in migration profiles between control cells and those expressing the respective variants. In the control experiments with wild-type RAC3, while a considerable number of GFP-positive cells were positioned in the intermediate zone, many cells advanced slowly toward the pial surface (Fig. 6A and B and Supplementary Video 3). In stark contrast, cells expressing RAC3-Q61L, -E62del, -D63N or -Y64C were stuck in the intermediate zone. When looking closer, cells expressing RAC3-E62del or -D63N remained round and appeared not to obtain the multipolar status (Fig. 6A and B and Supplementary Videos 4 and 5). On the other hand, cells expressing RAC3-Q61L or -Y64C were observed to transform into the multipolar shape but then fail to undergo the multipolar-bipolar transition (Fig. 6A and B and Supplementary Videos 6 and 7). Quantification analyses revealed that migration distance of the variant-expressing neurons at the intermediate zone–cortical plate boundary was much shorter than that of the control cells (Fig. 6C). The average migration speed of neurons expressing each *RAC3* variant was also reduced when compared to that of control cells (Fig. 6D).

### Role of PAK1 as a downstream effector of RAC3 in neuronal migration *in vivo*

**Figure 7:**
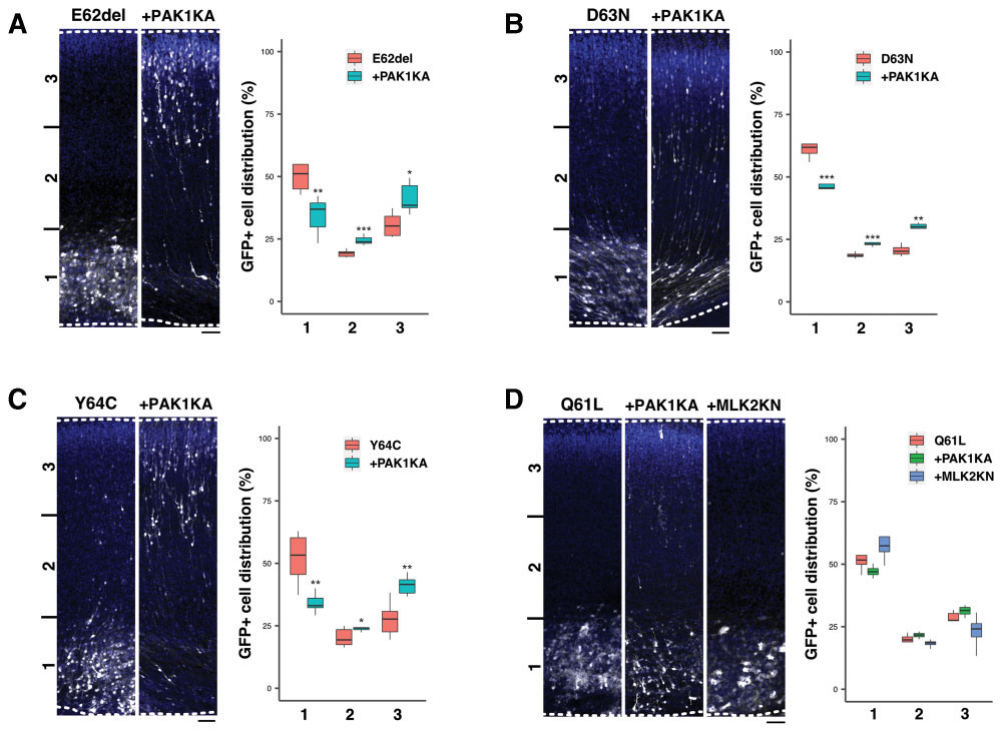
**Rescue effects by a dominantly negative PAK1 on migration defects caused by the four variants in the Switch II region.** pCAG-Myc-RAC3-E62del (**A**), -D63N (**B**) or -Y64C (**C**) (0.1 μg each) was co-electroporated with pCAG-EGFP (0.5 μg) together with pCAG-Flag vector (1.0 μg, control) (*left*) or pCAG-Flag-PAK1KA (1.0 μg) (*right*). (**D**) pCAG-Myc-RAC3-Q61L (0.1 μg) was co-electroporated with pCAG-EGFP (0.5 μg) together with 1.0 μg each of pCAG-Flag vector (control) (*left*), pCAG-Flag-MLK2KN (*middle*) or pCAG-Flag-PAK1KA (*right*). Analysis was done as in Fig. 5. Quantification results of the distribution of GFP-positive neurons in distinct regions (bin 1–3) of the cerebral cortex were shown in boxplot for each condition. Number of replicates, *n* ≥ 5. The significance of difference between control and each rescue condition was determined using Welch’s *t*-test (**A**–**C**) or Dunnett’s test (**D**), and shown in boxplot. (**A**, bin 1) E62del versus +PAK1KA, *P* = 0.005591. (**A**, bin 2) E62del versus +PAK1KA, *P* = 0.0006076. (**A**, bin 3) E62del versus +PAK1KA, *P* = 0.01177. (**B**, bin 1) D63N versus +PAK1KA, *P* = 0.0007075. (**B**, bin 2) D63N versus +PAK1KA, *P* = 0.0009054. (**B**, bin 3) D63N versus +PAK1KA, *P* = 0.001021. (**C**, bin 1) Y64C versus +PAK1KA, *P* = 0.005963. (**C**, bin 2) Y64C versus +PAK1KA, *P* = 0.04256. (**C**, bin 3) Y64C versus +PAK1KA, *P* = 0.003158. (**D**, bin 1) Q61L versus +PAK1KA, *P* = 0.338; Q61L versus +MLK2KN, *P* = 0.807. (**D**, bin 2) Q61L versus +PAK1KA, *P* = 0.311; Q61L versus +MLK2KN, *P* = 0.993. (**D**, bin 3) Q61L versus +PAK1KA, *P* = 0.393; Q61L versus +MLK2KN, *p* = 0.673. ***P < 0.001, **P < 0.01, *P < 0.05; Scale bar = 50 μm.

Since PAK1 is a downstream effector for RAC3 and activating *PAK1* variants cause NDD,^45^ dysregulation of this kinase is most likely to be involved in the pathogenesis of *RAC3* variants. We thus investigated the possible involvement of PAK1 in the migration defects caused by the four *RAC3* variations localized in the Switch II region. When pCAG-EGFP was co-electroporated with pCAG-Myc-RAC3-D63N, -E62del or -Y64C, together with pCAG-Flag-PAK1KA encoding a kinase-negative version of PAK1, the positional defects of GFP-positive cells were partially rescued at P0 (Fig. 7A–C). The observed rescue effects by PAK1KA confirmed that these three variants cause a hyper-activation of PAK1, which is supposed to be a crucial pathogenic mechanism of *RAC3*-related disorder caused by these three variations. To confirm the variant-mediated activation of PAK1, these three variants were expressed with PAK1 in COS7 cells, or without PAK1 in primary cultured cortical neurons. Consequently, activation of exogenous and endogenous PAK1 was observed in COS7 cells and cortical neurons, respectively (Supplementary Fig. 9A, B, D and E). In addition, when these variants were electroporated into embryonic mice brains, endogenous PAK1 activation was again detected in cortical neurons (Supplementary Fig. 9C and F). On the other hand, PAK1KA had no effects on the migration defects produced by RAC3-Q61L under the same experimental conditions (Fig. 7D). This result implicates the possibility that other downstream effector(s) is also involved in the pathogenicity of *RAC3*-related disorder. To answer this question, we selected another kinase MLK2, an activator for broad MAP kinase cascades including JNK (c-Jun N-terminal kinase), ERK (extracellular signal-regulated kinase) and p38,^47^ as the next candidate effector molecule involved in RAC3-Q61L-mediated pathophysiological mechanism. However, MLK2 failed to rescue the aberrant migration phenotype by RAC3-Q61L (Fig. 7D), indicating that yet unidentified downstream effector(s) plays a crucial role in the variation-mediated signalling dysregulation.

### Effects of the four *RAC3* variants in the Switch II region on axon development during corticogenesis

**Figure 8:**
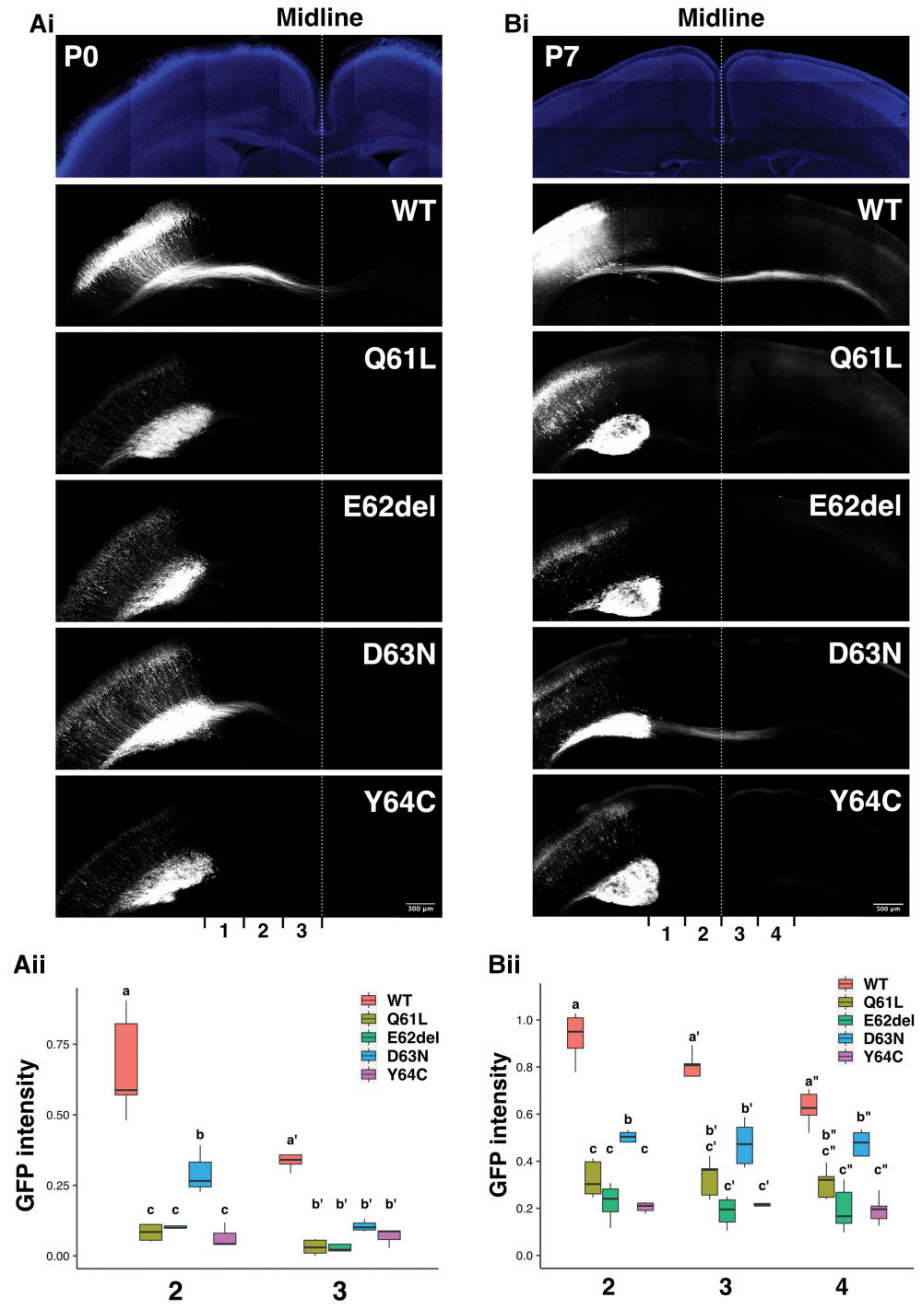
**Role of the four disease-causative variants in the Switch II region in the axon growth *in vivo.*** [**A**(**i**) and **B**(**i**)] pCAG-GFP was co-electroporated with pCAG-Myc-RAC3 (wild-type, WT), -RAC3-Q61L, -E62del, -D63N or -Y64C (0.1 μg each) into the ventricular zone progenitor cells at E14.5. Coronal sections were prepared at P0 [**A**(**i**)] or P7 [**B**(**i**)], and stained with anti-GFP (white). DAPI staining (blue) of a slice was also shown (*top*). Scale bars = 300 μm [**A**(**i**)] and 500 μm (C). [**A**(**ii**) and **B**(**ii**)] The GFP intensity of the callosal axon was measured at P0 [**A**(**ii**)] or P7 [**B**(**ii**)] in different regions [bins 1–3 for **A**(**i**) and bins 1–4 for **B**(**i**)], and then the relative intensities of bins were normalized with bin 1 as 1.0. Number of replicates, *n* ≥ 4. Different letters above bars represent significant differences, *P* < 0.05, according to a Tukey’s test. [**A**(**i**), bin 2] Q61L versus WT, *P* < 0.001; E62del versus WT, *P* < 0.001; D63N versus WT, *P* < 0.001; Y64C versus WT, *P* < 0.001; E62del versus Q61L, *P* = 1.0000; D63N versus Q61L, *P* = 0.0205; Y64C versus Q61L, *P* = 0.9756; D63N versus E62del, *P* = 0.0444; Y64C versus E62del, *P* = 0.9835; Y64C versus D63N, *P* = 0.0235. [**A**(**i**), bin 3] Q61L versus WT, *P* < 0.001; E62del versus WT, *P* < 0.001; D63N versus WT, *P* < 0.001; Y64C versus WT, *P* < 0.001; E62del versus Q61L, *P* = 0.997; D63N versus Q61L, *P* = 0.646; Y64C versus Q61L, *P* = 0.997; D63N versus E62del, *P* = 0.537; Y64C versus E62del, *P* = 0.976; Y64C versus D63N, *P* = 0.936. [**B**(**i**), bin 2] Q61L versus WT, *P* < 0.001; E62del versus WT, *P* < 0.001; D63N versus WT, *P* < 0.001; Y64C versus WT, *P* < 0.001; E62del versus Q61L, *P* = 0.1794; D63N versus Q61L, *P* = 0.0132; Y64C versus Q61L, *P* = 0.0755; D63N versus E62del, *P* < 0.001; Y64C versus E62del, *P* = 0.9784; Y64C versus D63N, *P* < 0.001. [**B**(**i**), bin 3] Q61L versus WT, *P* < 0.001; E62del versus WT, *P* < 0.001; D63N versus WT, *P* < 0.001; Y64C versus WT, *P* < 0.001; E62del versus Q61L, *P* = 0.31921; D63N versus Q61L, *P* = 0.38265; Y64C versus Q61L, *P* = 0.51491; D63N versus E62del, *P* = 0.00505; Y64C versus E62del, *P* = 0.99767; Y64C versus D63N, *P* = 0.01394. [**B**(**i**), bin 4] Q61L versus WT, *P* < 0.001; E62del versus WT, *P* < 0.001; D63N versus WT, *P* = 0.01014; Y64C versus WT, *P* < 0.001; E62del versus Q61L, *P* = 0.48422; D63N versus Q61L, *P* = 0.34330; Y64C versus Q61L, *P* = 0.45377; D63N versus E62del, *P* = 0.00869; Y64C versus E62del, *P* = 0.99996; Y64C versus D63N, *P* = 0.00904.

Anomalies of corpus callosum in subjects with *RAC3*-related disorder, such as hypoplasia and agenesis, reflect defects in axon growth (Fig. 1C). Specifically, an anomaly at the level of the splenium strongly suggests impaired axon elongation of pyramidal neurons in layer II/III and V of the temporal, parietal and occipital lobes. In this context, the *in utero* electroporation method is available to examine axon elongation from pyramidal neurons in layer II/III in the parietal lobe during mouse brain corticogenesis. When the wild-type of RAC3 (as a control), RAC3-Q61L, -E62del, -D63N or -Y64C were introduced into the ventricular zone progenitor cells at E14.5 and axon bundle of corpus callosum was visualized at P0, neurons expressing RAC3-Q61L, -E62del and -Y64C did not project axons under the conditions where control axon bundle reached the midline [Fig. 8A(i) and (ii)]. On the other hand, axon elongation was observed but resulted in significantly delayed in neurons expressing RAC3-D63N [Fig. 8A(i) and (ii)]. When we further analysed the long-term effects at P7, control neurons extended the axon bundle into the contralateral white matter, whereas axon elongation could not be detected yet for cells expressing RAC3-Q61L, -E62del and -Y64C [Fig. 8B(i) and (ii)]. Notably, although axons from the hemisphere containing RAC3-D63N-expressing cells eventually reached the contralateral white matter at P7, the bundle was observed to be thinner compared to the control neurons [Fig. 8(i)]. Then, rescue effects by dominant negative PAK1 on the defects in axon growth were examined *in vivo*. When PAK1KA was co-expressed with RAC3-Q61L, -E62del, -D63N or -Y64C, the defective phenotypes were at least partially rescued for RAC3-E62del, -D63N or -Y64C (Supplementary Fig. 10). In contrast, the phenotype by RAC3-Q61L was not rescued, as in the case of cortical neuron migration (Supplementary Fig. 10). These results strongly suggest that abnormal PAK1 activation contributes to the impaired axon elongation by p.E62del, p.D63N and p.Y64C variations, which leads to the corpus callosum dysgenesis, while yet unidentified effector(s) is involved in the axon phenotype by p.Q61L variation. Further analyses are required to determine if the observed impaired axon growth is a primary phenotype or secondary to migration defects.

## Discussion

We identified seven distinct *de novo* missense variants (five novel, one recurrent) and a novel *de novo* in-frame deletion in *RAC3* in 10 patients presenting with NEDBAF, which is a complex syndromic NDD characterized by a moderate to severe psychomotor delay leading to intellectual disability, associated with peculiar neurological and extra-neurological features. The potential contribution of pathogenic copy number variants to the reported phenotypes was excluded in all subjects (Supplementary Table 1). Regarding the neuroimaging features of *RAC3* deficiency in this study, the most frequent findings were abnormalities in corpus callosum and cortical development, collectively noted in 100% and 90% of the individuals reported so far, respectively (Supplementary Table 2). In particular, the corpus callosum was found to be frequently dysplastic, with thinner splenium and/or a thick genu, indicating the presence of axonal growth anomalies. On the other hand, cortical malformations included diffuse dysgyria, polymicrogyria and small grey matter heterotopias, showing remarkable evidence of abnormal neuronal migration. Although disruption of corticogenesis has been presumed to be crucial for the pathogenesis and pathophysiology of *RAC3*-related disorder, the underlying molecular mechanisms remained unknown. We thus investigated the pathophysiological significance of *RAC3* variants causing NEDBAF during cortical development *in vitro* and *in vivo*.

We assume that the pathogenic effects of the seven variants in the Switch II region (p.A59G, p.G60D, p.Q61L, p.E62K, p.E62del, p.D63N and p.Y64C) are related to the aberrant interactions with the regulatory proteins in variant- and context-dependent manners, since the region is essential for the physical association with GEFs and GAPs. Being positioned in the effector-binding loop (aa32–41), which is overlapping with the Switch I region, the p.P34R variant might impact the interaction with downstream effectors as well as GEFs and GAPs. Indeed, when compared to other variants, RAC3-P34R displayed weaker affinities to RBRs of all effector molecules tested (Fig. 4A). Notably, the patient harbouring p.P34R presented syntelencephaly, a rare brain malformation characterized by an abnormal midline connection of the cerebral hemispheres, suggesting a role of RAC3 in the cleavage process of prosencephalon.^19^ Meanwhile, p.G12R and p.K116N affect residues conserved in most small GTPases within the G1 and G4 boxes, respectively. In line with the different roles of these boxes in the interaction with guanine nucleotides, these two variants may affect biochemical properties distinctly. Indeed, while the p.G12R variation activated the protein by suppressing GTP-hydrolysis activity, p.K116N facilitated GTP/GDP-exchange reaction. Collectively, biochemical analyses strongly suggest that the 11 *RAC3* variants analysed show ‘gain-of-function’ phenotypes; they preferentially bind GTP in variant type-specific modes and are hyper-activated in various degrees. This hypothesis is consistent with the results that all the *RAC3* variants exhibited lamellipodia formation with cell rounding when overexpressed in primary cultured hippocampal neurons.

Although the 11 *RAC3* variants are supposed to be activated, it is surprising that these variants displayed different affinities to RBRs of various downstream effectors, including PAK1, MLK2, IRSp53, N-WASP, ROCK and RTKN. Pull-down assay with PAK1-RBR has so far been conducted to assess the ‘activation state’ of RAC by measuring the amount of the GTP-bound form co-precipitated, under the tacit recognition that the interaction with an RBR of any effector molecule precisely reflects the GTP-bound activated state. However, the results obtained here indicate that the RAC3-GTP amount precipitated by the RBR of PAK1 or other effectors was not necessarily correlated with the activation state of RAC3. We thus suggest that disease-causative *RAC3* variants even within the same structural/functional domains differently modulate the activation state of RAC3, and show specific spectra in the interaction with effectors, leading to abnormal upregulation of relevant effectors in variant type and context-dependent manners. The hyperactivation of certain signalling pathways may underlie the pathogenesis and pathophysiology of the divergent clinical features observed in each patient. Meanwhile, each variant is possible to not only activate particular downstream effectors differently but also exert dominant negative effects on some other signalling pathways simultaneously. In any case, intracellular signalling networks should be affected by each variant both in a qualitatively and quantitatively different manner, which may contribute to the variable phenotypes observed in patients with *RAC3*-related disorder.

To clarify the pathophysiological significance of *RAC3* variations *in vivo*, we focused on p.D63N, p.E62del, p.Y64C and p.Q61L in the Switch II region, a variation hotspot not only for *RAC3* but also for *RAC1* and *CDC42*. Although these four variants should affect different signalling pathways *in vitro*, their expression in cortical neurons resulted in similar phenotypes *in vivo*: severe defects in neuronal migration, mispositioning, and eventual cluster formation in the intermediate and subventricular zones, as well as defects in axon extension to the contralateral hemisphere during corticogenesis. As for the migration phenotypes, time-lapse imaging revealed differences among the analysed variants. Newborn neurons expressing RAC3-Q61L or -Y64C appeared to become multipolar but did not transform into the bipolar status, whereas cells expressing RAC3-E62del or -D63N failed to become even multipolar. Further analyses are required to elucidate the pathophysiological meaning of this phenotypic difference. Cluster formation by mislocalized NeuN-positive neurons should account for the pathogenic mechanisms underlying the heterotopia and, partially, polymicrogyria and dysgyria, which represent the main neuronal migration/positioning abnormality observed in *RAC3*-related disorder. Also, considering the corpus callosum anomalies observed in affected individuals, it is a reasonable conclusion that the four *RAC3* variants suppress axon elongation *in vivo*. The partial suppression of axon growth by RAC3-D63N may explain the absence of ‘white matter thinning’ in the individual harbouring this variant.

Since activating *PAK1* variants have been shown to cause NDDs,^45^ dysregulation of PAK1 may be involved in the migration defects associated with the *RAC3* variants. Indeed, the abnormal phenotypes caused by p.D63N, p.E62del and p.Y64C were rescued by a kinase-negative version of PAK1, PAK1KA. We thus concluded that these three variants hyper-activated PAK1 and provoked defects in neuronal migration during corticogenesis, irrespective of their different affinities to PAK1 *in vitro*. On the other hand, despite the strong affinity, PAK1 did not appear to be related to the RAC3-Q61L-mediated migration defects, since PAK1KA did not exert rescue effects. These results strongly suggest that PAK1 is not the sole molecule regulating cortical neuron migration downstream of RAC3.

*De novo RAC1* missense variants have also been identified in NDDs with global developmental delay/intellectual disability and brain size abnormalities as core phenotypes (Mental Retardation autosomal dominant 48, MRD48, OMIM 617751).^16^ While Rac3 is mainly, if not exclusively, expressed in developing and adult neurons, Rac1 is ubiquitously expressed from the early embryonic stage. Despite the different expression profiles, the precise functional difference between RAC1 and RAC3 has not been investigated. Rather, RAC1–3 have seemingly been equated in various analyses, since they show very similar phenotypes *in vitro*, especially when overexpressed.^60^ RAC1 and RAC3 are primarily divergent in the last nine carboxy-terminal residues, which include a polybasic region and an adjacent CAAX box, where the C is a Cys, the two A residues are aliphatic amino acids and the X can be any residue.^5^ The CAAX box is posttranslationally modified and is crucial, together with the polybasic region, for the subcellular localization of the protein.^61^ Given the highly homologous structure and common effector molecules, the differences in the pathogenic mechanisms and clinical phenotypes associated with *RAC1* and *RAC3* variants may be attributable to diverse subcellular distribution, at least partially. We assume that differentially distributed *RAC1* and *RAC3* variants may spatiotemporally hyper-activate interactable effectors in context-dependent manners.

The *RAC1* missense variants associated with MRD48 exert their pathophysiological functions differently from *RAC3*.^16^ The p.Y64D variant was determined to be constitutively activated, since its expression in fibroblasts resulted in a rounding shape and formation of lamellipodia.^16^ The p.C18Y and p.N39S variants were instead considered as dominant negative alleles, based on fibroblast morphology analyses and *in vivo* zebrafish analyses.^16^ Meanwhile, three additional variants (p.V51M, p.P73L and p.C157Y) were categorized as neither active nor inactive versions and considered to have context-dependent effects.^16^ Although GTP/GDP-binding states were not determined biochemically, these results show that (i) the variant position may determine the activation state of RAC1, ranging from dominant negative to neutral to constitutively active; and (ii) the precise control of RAC1 activity is crucial for correct brain development. In this context, pathogenic variants in *TRIO*, encoding a GEF which activates RAC1 through the first GEF domain (GEFD1), were shown to cause neurodevelopmental impairment, behavioural disturbances, microcephaly or macrocephaly and skeletal features [autosomal dominant intellectual developmental disorder with microcephaly (MRD44, OMIM 617061) or macrocephaly (MRD63, OMIM 618825)].^62,63^ Affected individuals with variations in *TRIO* present with variable neurodevelopmental phenotypes. For example, missense changes affecting the GEFD1 domain were found to inhibit TRIO function and thereby inactivate RAC1. In contrast, missense variations in the seventh spectrin domain of TRIO, a variation hotspot, resulted in RAC1 hyperactivation, further supporting the relevance of the tight control of RAC1 signalling during brain development.^64^

Unlike the case of RAC1, we concluded that all the 11 tested *RAC3* variants act as GTP-bound active versions in varying degrees. From the results obtained in this and previous studies, the variant-dependent spatiotemporal dysregulation (hyper-activation or inhibition) of subsets of common downstream effectors appears to cause diverse phenotypic features in *RAC1*- and *RAC3*-related disorders. Given that variants affecting RAC3 function cause polymicrogyria, heterotopia and dysgyria, which are only occasional in *RAC1*-related disorder,^16^ we assume that RAC3 plays a central role in neuronal migration *in vivo*, although Rac3 knockout mice displayed little phenotype in terms of cortical architecture, perhaps due to compensation by Rac1.^65^ In contrast, *RAC1* variants are associated with abnormalities in brain size (extraordinary spread from −5 to +4.5 standard deviation of occipital-frontal circumference), which are rare among *RAC3* patients, suggesting a major role of RAC1 in neurogenesis and/or apoptosis.^15,16^ Since the disruption of synaptic functions is involved in the pathogenesis of global developmental delay/intellectual disability, RAC1 and RAC3 are supposed to cooperate to build up the neuronal signalling network and functions.

Another Rho family protein CDC42 is essential for the control of cell polarity, migration, endocytosis, and cell cycle.^66^*De novo* missense variations in *CDC42* have been reported in patients with clinically heterogeneous but overlapping phenotypes (Takenouchi-Kosaki syndrome, OMIM 616737).^35,67,68^ Biochemical assays revealed that all the variants were activated as in the case of *RAC3* variants.^35,36^ Considering that CDC42 and RAC proteins are strictly linked in their physiological functions through a variety of shared downstream effectors, it is plausible that the clinical features of *CDC42*-related disorder overlap with those of *RAC1*- and *RAC3*-related conditions. The intersection of the pathogenic mechanisms underlying *RAC1*-, *RAC3*-, *CDC42*-, and *TRIO*-related disorders is reflected in their overlapping clinical phenotypes (Supplementary Fig. 1^1^).

In the present study, we identified six novel *de novo* pathogenic variants in *RAC3* in unrelated individuals with NEDBAF. A variable degree of biochemically activated states was observed for the novel and previously reported RAC3 variants tested. Activation of these variants was also confirmed biologically with primary hippocampal neurons. It is noteworthy that each variant displayed different affinities to a variety of effector molecules *in vitro*, which may dysregulate downstream cellular pathways in variant-specific manners and contribute to the pathogenesis of patient-specific clinical features as well as common ones. We then performed *in vivo* analyses of four variants (p.D63N, p.E62del, p.Y64C and p.Q61L) affecting the Switch II region, a variation hot spot. They prevented neuronal migration during corticogenesis, through dysregulation of PAK1-mediated signalling pathway in some cases. It is notable that the abnormally positioned neurons eventually formed clusters in the ventricular and subventricular zones in developing cerebral cortex, which may be an underlying mechanism of heterotopia, polymicrogyria and dysgyria. These four variations also caused defective axon development *in vivo,* explaining their involvement in corpus callosum hypoplasia/agenesis. Collectively, we conclude that all the *RAC3* variants identified so far are gain-of-function, and variation-dependent activation of downstream signalling pathways may contribute to the heterogeneous phenotypes observed in individuals with *RAC3*-related disorder. The results obtained in this study contribute to the understanding of the molecular machinery underlying this complex condition, providing insights for the development of novel targeted drugs aimed at interfering with the disease pathogenesis.

## Web resources

ClinVar; https://www.ncbi.nlm.nih.gov/clinvar

Combined Annotation Dependent Depletion (CADD); http://cadd.gs.washington.edu

DECIPHER; https://decipher.sanger.ac.uk

Gene Cards; https://www.genecards.org

Gene Matcher; http://www.genematcher.org

Genome Aggregation Database (GnomAD); http://gnomad.broadinstitute.org

Genomic Evolutionary Rate Profiling (GERP); http://mendel.stanford.edu/SidowLab/downloads/gerp/

Mutation Taster; http://www.mutationtaster.org NeXtProt; https://www.nextprot.org

Online Mendelian Inheritance in Man; https://www.ncbi.nlm.nih.gov/Omim

PubMed; https://www.ncbi.nlm.nih.gov/pubmed

RefSeq; https://www.ncbi.nlm.nih.gov/refseq

SIFT; https://sift.bii.a-star.edu.sg

Simons Foundation Autism Research Initiative: SFARI; https://www.sfari.org

UniProt; https://www.uniprot.org

UCSC Human Genome Database; https://www.genome.ucsc.edu

Varsome; https://varsome.com

Ensembl Variant Effect Predictor (VEP); https://www.ensembl.org/info/docs/tools/vep/index.html

## Supplementary Material

awac106_Supplementary_Data

## Appendix 1

A full list of members and their affiliations appears in the Supplementary material.

## Undiagnosed Disease Network

Gabrielle Brown, Manish J. Butte, Esteban C. Dell'Angelica, Naghmeh Dorrani, Emilie D. Douine, Brent L. Fogel, Irma Gutierrez, Alden Huang, Deborah Krakow, Hane Lee, Sandra K. Loo, Bryan C. Mak, Martin G. Martin, Julian A. Martínez-Agosto, Elisabeth McGee, Stanley F. Nelson, Shirley Nieves-Rodriguez, Christina G.S. Palmer, Jeanette C. Papp, Neil H. Parker, Genecee Renteria, Janet S. Sinsheimer, Jijun Wan, Lee-kai Wang and Katherine Wesseling Perry.

## Telethon Undiagnosed Diseases Program

Vincenzo Nigro, Nicola Brunetti-Pierri, Giorgio Casari, Gerarda Cappuccio, Annalaura Torella, Michele Pinelli, Francesco Musacchia, Margherita Mutarelli, Diego Carrella, Giuseppina Vitiello, Valeria Capra, Giancarlo Parenti, Vincenzo Leuzzi, Angelo Selicorni, Silvia Maitz, Sandro Banfi, Marcella Zollino, Mario Montomoli, Donatelli Milani, Corrado Romano, Albina Tummolo, Daniele De Brasi, Antonietta Coppola, Claudia Santoro, Angela Peron, Chiara Pantaleoni, Raffaele Castello and Stefano D’Arrigo.

## Abbreviations

GAP =: GTPase-activating protein
GEF =: guanine nucleotide-exchange factor
GTPase =: guanosine triphosphatase
NDD =: neurodevelopmental disorder
NEDBAF =: neurodevelopmental disorder with structural brain anomalies and dysmorphic facies
RBR =: RAC-binding region
SVZ =: subventricular zone
