# Supplementary material for: Variant-specific changes in RAC3 function disrupt corticogenesis in neurodevelopmental phenotypes
Source: Brain. 2022 Jul 4;145(9):3308–27. doi: 10.1093/brain/awac106 (PMC9473360; doi:10.1093/brain/awac106)
Supplement: awac106_Supplementary_Data [file awac106_supplementary_data.zip › awac106 Supplementary Material.pdf]

Variant-specific changes in RAC3 function disrupt corticogenesis in neurodevelopmental phenotypes

Marcello Scala,† Masashi Nishikawa,† Hidenori Ito,† Hidenori Tabata, Tayyaba Khan, Andrea Accogli, Laura Davids, Anna Ruiz, Pietro Chiurazzi, Gabriella Cericola, Björn Schulte, Kristin G. Monaghan, Amber Begtrup, Annalaura Torella, Michele Pinelli, Anne-Sophie Denommé-Pichon, Antonio Vitobello, Caroline Racine, Maria Margherita Mancardi, Courtney Kiss, Andrea Guerin, Wendy Wu, Elisabeth Gabau Vila, Bryan C. Mak, Julian A. Martinez-Agosto, Michael B. Gorin, Bugrahan Duz, Yavuz Bayram, Claudia M. B. Carvalho, Jaime E. Vengoechea, David Chitayat, Tiong Yang Tan, Bert Callewaert, Bernd Kruse, Lynne M. Bird, Laurence Faivre, Marcella Zollino, Saskia Biskup, Undiagnosed Diseases Network, Telethon Undiagnosed Diseases Program, Pasquale Striano, Vincenzo Nigro, Mariasavina Severino, Valeria Capra, Gregory Costain and Koh-ichi Nagata

†These authors contributed equally to this work.

Supplementary Material

1. Supplementary Methods

2. Supplementary Results

3. Supplementary Figures

4. Supplementary Videos

5. Supplementary References

6. Supplementary Affiliations

1. Supplementary Methods

Participants enrolment

The patients investigated in this study were enrolled at several different international research centers and hospitals: Centre de Génétique et Centre de Référence Anomalies du Développement et Syndromes Malformatifs de l'interrégion Est, Hôpital d'Enfants, CHU de Dijon, France; Department of Human Genetics, Emory Healthcare, USA; Departments of Human Genetics, Pediatrics and Psychiatry, David Geffen School of Medicine at UCLA, USA; Department of Pediatrics, University of California San Diego, USA; Genetics/Dysmorphology, Rady Children's Hospital San Diego, USA; Dipartimento Universitario Scienze della Vita e Sanità Pubblica, Università Cattolica Sacro Cuore, Rome, Italy; Division of Clinical and Metabolic Genetics, Department of Pediatrics, The Hospital for Sick Children, Canada; Genetica Medica, Fondazione Policlinico Universitario A. Gemelli IRCCS, Italy; Neuropediatric Department Helios-Klinikum Hildesheim Hildesheim, Germany; Parc Taulí Hospital Universitari, Institut d'Investigació i Innovació Parc Taulí I3PT, Universitat Autònoma de Barcelona, Spain; Unit of Child Neuropsychiatry, IRCCS Istituto Giannina Gaslini, Italy.

Genetic testing and data analysis

After standard DNA extraction, trio-ES (#1-3, #8, and #10), singleton exome sequencing (ES) (#4, #6, and #9), trio-genome sequencing and RNA sequencing (#5), or intellectual disability next-generation sequencing gene panel (480 genes) (#7) were performed. NGS panels and ES were carried out as previously described (Aspromonte *et al.,* 2019; Bowling *et al.*, 2017; Harripaul *et al.*, 2017; Murdock *et al.*, 2021; Tarailo-Graovac *et al.*, 2016). QC statistics with FastQC (http://www.bioinformatics.bbsrc.ac.uk/projects/fastqc) was used to assess the quality of the sequence reads. Reads alignment to the reference human genome (hg19, UCSC assembly, February 2009) was performed through BWA with default parameters (Li and Durbin, 2009). Recalibration of the quality score and for indel realignment and variant calling was performed through the HaplotypeCaller algorithm within the GATK package (DePristo *et al.*, 2011; McKenna *et al.*, 2010). Variants were annotated with ANNOVAR (Wang *et al.*, 2010). After being filtered out for minor allele frequency (MAF) ≤ 0.01 in genomic databases (GnomAD, https://gnomad.broadinstitute.org), the predicted impact of candidate variants on protein structure and function was evaluated through *in silico* tools, which included:

Combined Annotation Dependent Depletion (CADD, https://cadd.gs.washington.edu), Mutation Taster (http://www.mutationtaster.org), Mutation Assessor (http://mutationassessor.org/r3/), Sorting Intolerant From Tolerant (SIFT, https://sift.bii.a-star.edu.sg), Polyphen-2 (http://genetics.bwh.harvard.edu/pph2/), and Human Splice Finder (http://umd.be/Redirect.html). Sanger sequencing was performed according to standard procedures (Tarailo-Graovac *et al.*, 2016) to confirm the most plausible candidate variants and for parental segregation analysis. Array Comparative Genomic Hybridization (Array-CGH) was performed in #1, #2, #4, #5, #6, #7, #8, and #10 as previously described (Redon *et al.*, 2009) and the detected rearrangements were interpreted according to the Decipher database (https://decipher.sanger.ac.uk). In patient #3, CNVs analysis was performed using XHMM software, which uses principal component analysis (PCA) normalization and a hidden Markov model to genotype CNVs from normalized read-depth information (Fromer *et al.*, 2012). Depth of coverage was calculated with GATK (Genome Analysis Toolkit, v3.5), and a matrix of the mean per-exon (RefSeq n = 193342) depth of coverage across all samples (n = 846) was obtained. The target read depths were first mean-centered and PCA was used on the read depth matrix to identify the components with high variance. These components, which explain most of the variation due to systematic noise, were subtracted from the matrix to obtain the normalized read depth matrix. In order to scale target depth values, XHMM used a z-score calculation to transform normalized read depths for each sample. The z-scores were then used to call CNVs with a hidden Markov model (HMM) algorithm to determine deletion or duplication regions based on below-average and above-average read depth. These calculations take into account the distance between exome targets, CNV rates and length distribution. CNVs were then filtered using the following criteria: estimated CNV length ≥1kb, XHMM quality score (SQ) ≥65, exons span >1, minor allele frequency (MAF) ≤1%. CNVs were annotated using RefSeq, (https://www.ncbi.nlm.nih.gov/refseq/), DGV17 and DECIPHER18 databases.

Antibodies

The following antibodies were used: anti-Rac3 (Novus Biologicals, Littleton, CO, Cat# NBP2-32058), anti-PAK1 (Cell Signaling Technology Cat# 2602, RRID:AB_330222), anti-phospho-PAK1 (Ser199/204) (Cell Signaling Technology Cat# 2605, RRID:AB_2160222), and anti-phospho-PAK1 (Thr423) (Cell Signaling Technology Cat# 2601, RRID:AB_330220).

2. Supplementary Results

Array-CGH

Patient #7 was found to harbor two rearrangements: arr[hg19] 17p13.3 (799610_912880) x 1 and Xp22.33 (1378391_1431726) x 3. The first deletion encompasses 4 OMIM genes (*ADAP1*, *608114; *COX19*, * 610429; *GET4*, *612056; *SUN1*, *607723), none of which is currently associated with a human phenotype. Furthermore, no patient with an overlapping rearrangement could be identified in the Decipher database (https://www.deciphergenomics.org/search/patients/results?q=7%3A799610-912880). The Xp22.33 duplication involves five genes, of which three OMIM (*ASMTL*, *300162; *IL3RA*, *430000; *SLC25A6*, *403000), but none associated with human disease so far. In the Decipher database, a single patient carried a partially overlapping rearrangement (ID 290028). Although reported to have seizures, this individual also carried two additional rearrangement and the Xp22.33 duplication (1378227_1387657) was interpreted as benign. Patient #8 harbored the maternally-inherited arr[hg19] 15q11.2 (22750082_23672560) x 1. This deletion encompasses 17 genes, including six OMIM genes (CYFIP1, *606322; MAGEL2, *605283; MKRN3, *603856; NIPA1, *608145; NIPA2, *608146; TUBGCP5, *608147). Three of these genes are morbid genes (*MAGEL2* - Schaaf-Yang syndrome, #615547; *MKRN3* - Precocious puberty, central, 2, # 615346; *NIPA1* - Spastic paraplegia 6, autosomal dominant, #600363), but no clinical feature consistent with the conditions associated with their haploinsufficiency could be identified either in the proband or in his healthy mother. The deleted interval is part of the 15q11.2 BP1–BP2 microdeletion syndrome, a rare condition characterized by developmental delay and neuropsychiatric features (Cox *et al.*, 2015; Rafi *et al.*, 2020). Since the deletion was inherited from the healthy mother, this rearrangement was not considered pathogenic in this individual, although a partial contribution to the neurobehavioral phenotype cannot be completely excluded due to the characteristic incomplete penetrance reported in patients with the 15q11.2 BP1–BP2 microdeletion syndrome. Patient #6 was found to harbor three maternally inherited duplication: arr[hg19] 3q12.2 (100348441_100438903) x3, 8p12 (29923503_29940466) x3, and 12p13.31 (7945545_7972180) x3. The first rearrangement (arr[hg19] 3q12.2 (100348441_100438903) x3) encompasses *GPR128* (OMIM * 612307) and *TFG* (* 602498). While *GPR128* is not a disease-causing gene, variants in *TFG* are associated with autosomal recessive spastic paraplegia 57 (OMIM # 615658) and Okinawa type hereditary motor and sensory neuropathy (OMIM # 604484). However, duplications involving TFG have not been linked to a clinicsl phenotype so far.

The second duplication (arr[hg19] 8p12 (29923503_29940466) x3) includes MIR548O2 (not OMIM) and *TMEM66* (OMIM * 614768), both not associated with a human disorder. The third rearrangement (arr[hg19] 12p13.31 (7945545_7972180) x3) encompasses *NANOG* (OMIM * 607937) and *SLC2A14* (OMIM * 611039), not associated with a human phenotype. According to the inheritance pattern and the involved genes, all these duplications are not predicted to have a pathogenic potential. Eventually, the array-CGH (patients #1, #2, #4, #5, and #10) and exome sequencing copy number variants (CNVs) analysis (patient #3) yielded negative results in the remaining cases.

Vineland Adaptive Behavior Scales, Third Edition (VinelandTM-3)

Vineland Adaptive Behavior Scales were administered to patients #4, #5, and #8. The overall level of adaptive functioning is described by the score on the Adaptive Behavior Composite (ABC), based on scores for three specific adaptive behavior domains: Communication, Daily Living Skills, and Socialization. The domain scores are also expressed as standard scores with a mean of 100 and standard deviation of 15. The Communication domain measures how well the patient listens and understands, expresses him/herself through speech, and reads and writes. The Daily Living Skills domain assesses the patient's performance of the practical, everyday tasks of living that are appropriate for his/her age. The individual's score for the Socialization domain reflects his/her functioning in social situations.

Patient #4 had an ABC of 59, which is well below the normative mean of 100, with a percentile rank for this overall score <1. Her Communication standard score was 55, corresponding to a percentile rank of <1. Her standard score for Daily Living Skills was 47, which corresponds to a percentile rank of <1 and represents a relative weakness for this individual. Her Socialization standard score was 64, corresponding to a percentile rank of 1 and representing a domain of relative strength for her.

Patient #5 had an ABC score of 39, which is well below the normative mean of 100. The percentile rank for this overall score is <1. Her Communication standard score was 34, which corresponds to a percentile rank of <1. Her standard score for Daily Living Skills was 27, which corresponds to a percentile rank of <1. This domain is a relative weakness for her, whereas socialization is a relative strength for this individual, with a standard score of 40 (percentile rank of <1).

Patient #8 had an ABC score of 63, which is well below the normative mean of 100. The percentile rank for this overall score is 1. His Communication standard score was 68, corresponding to a percentile rank of 2 and representing a relative strength for him. His standard score for Daily Living Skills was 56, which corresponds to a percentile rank of <1. This domain is a relative weakness for him, such as his Socialization, due to a standard score of 58 (percentile rank of <1).

3. Supplementary Figures

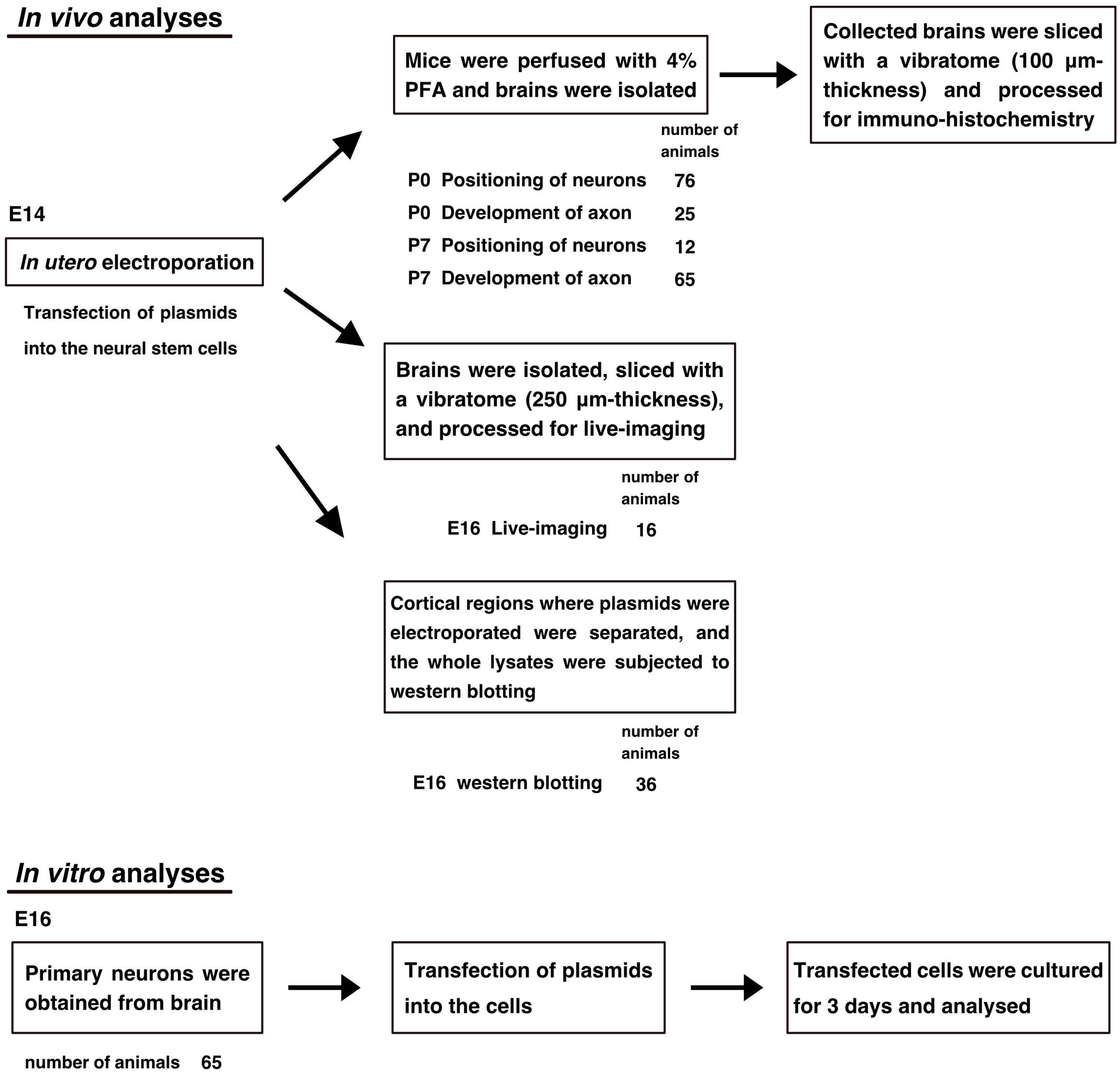

Supplementary Figure 1. Graphical time-line of the study design

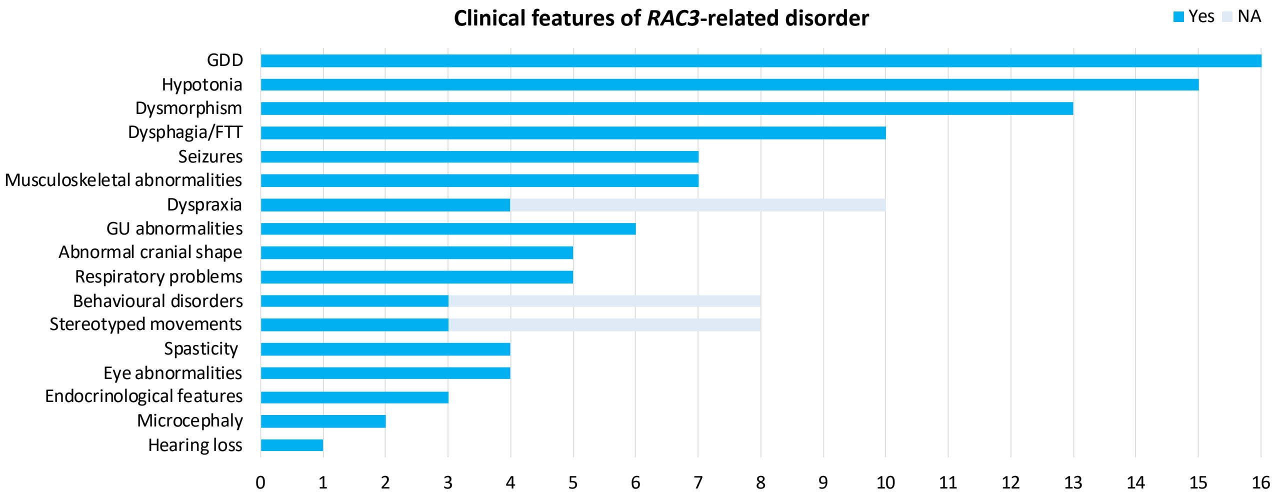

Supplementary Figure 2. Phenotypic spectrum of RAC3-related disorder. Bar graph illustrating the distribution of clinical features in the whole population of *RAC3* patients.

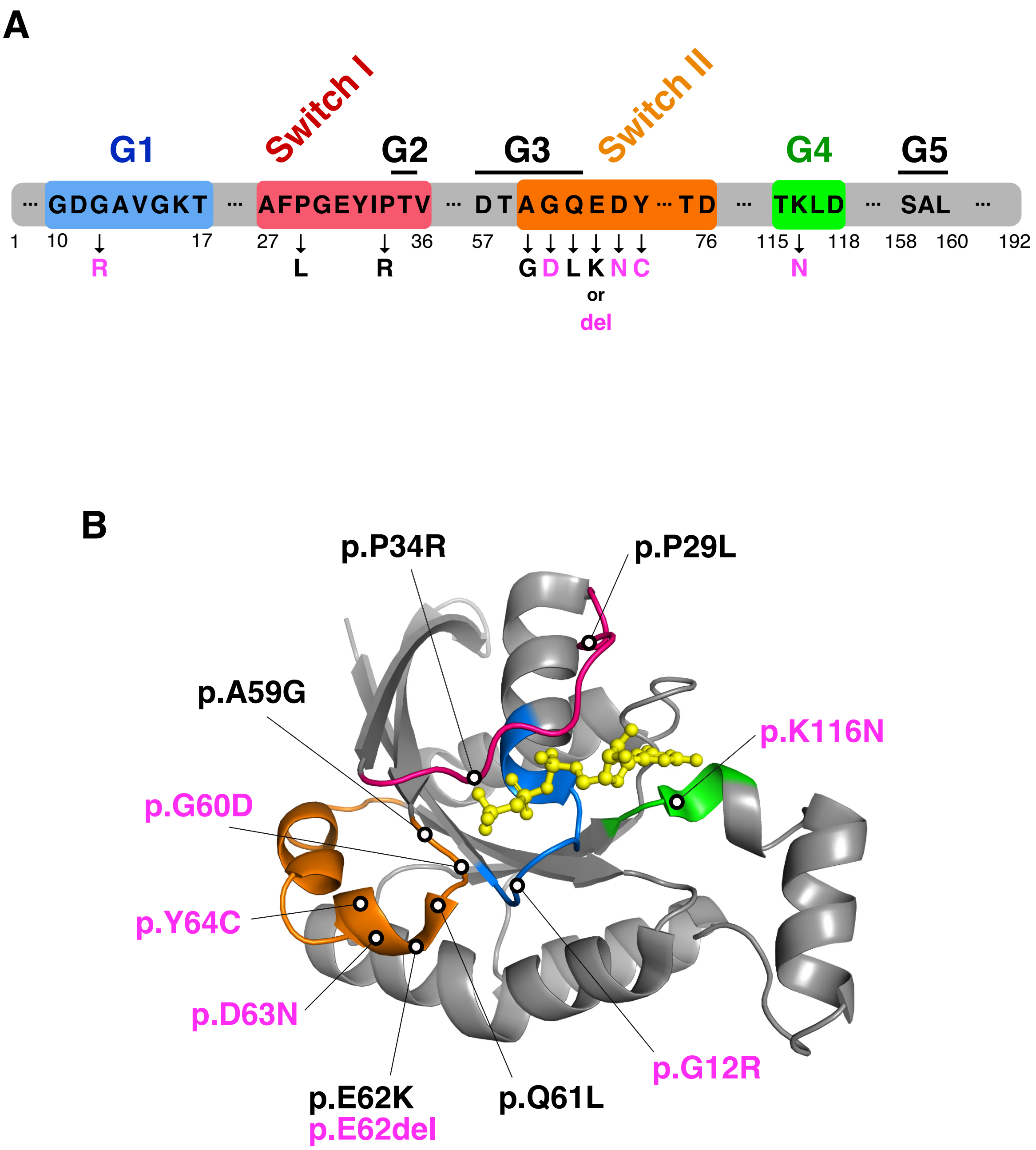

Supplementary Figure 3. Schematic representation of RAC3 variants. (A) Schematic representation of RAC3 structure was shown with the positions of the variations identified in this study (p.G12R, p.G60D, p.E62del, p.D63N, p.Y64C, and p.K116N) in magenta, and previously (p.P29L, p.P34R, p.A59G, p.Q61L, and p.E62K) in black. **(B)** The 3D structure of GTP-bound RAC3 was predicted with the SWISS-MODEL program, based on the structure of RAC1 (PDB ID: 1E96). Structural domains are colored as follows: G1, blue; Switch I, red; Switch II, orange; G4, green. G2, G3 and G5 boxes are shown with black bars in (A). GTP is indicated in yellow in (B).

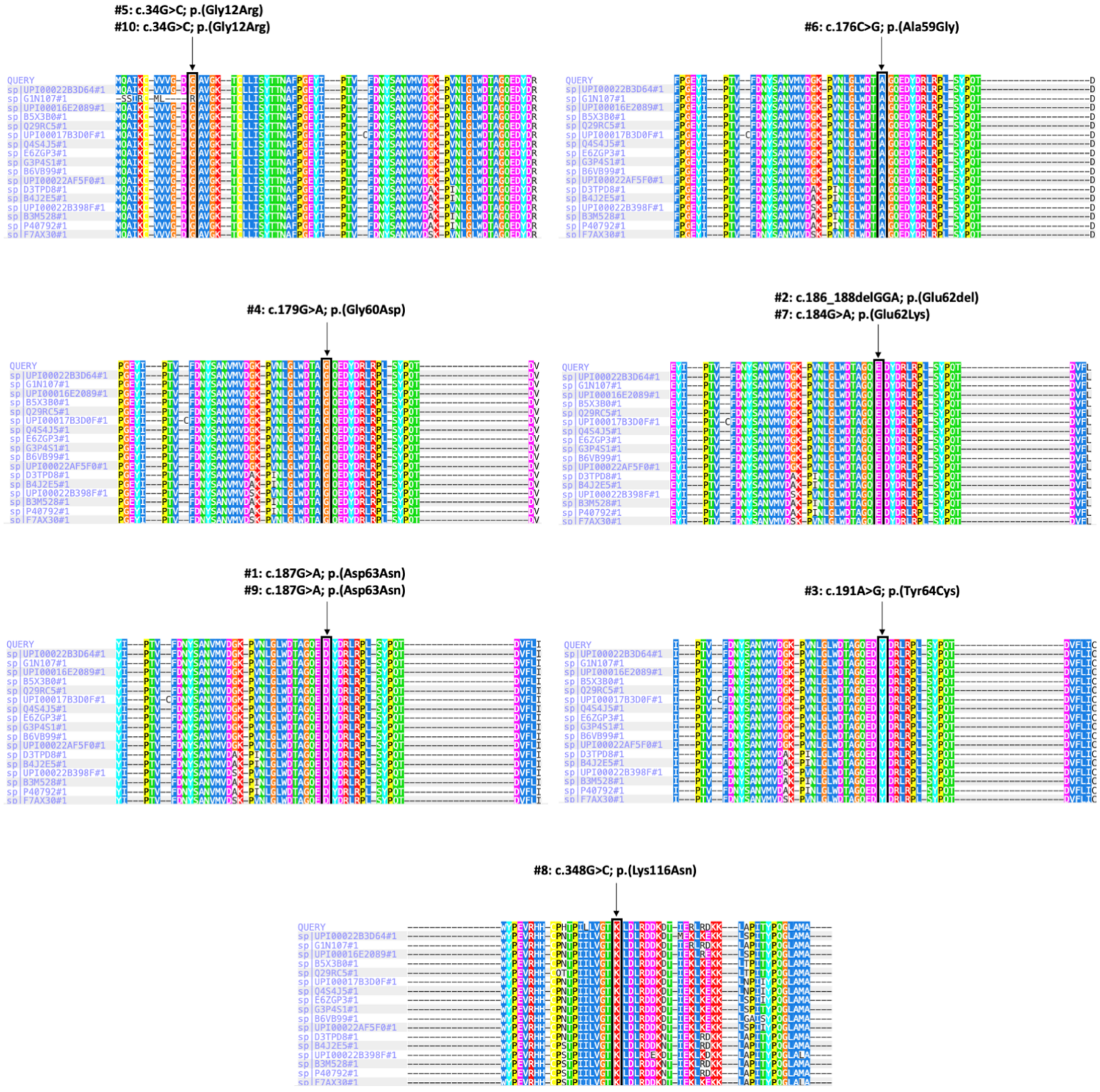

Supplementary Figure 4. Conservation among different species of the RAC3 residues affected in the reported patients. *RAC3* variants are reported according to the NM_005052.3 transcript. UPI00022B3D64 (A0A087XGA4): Poecilia formosa (Amazon molly) (Limia formosa); G1N107_MELGA: Meleagris gallopavo (Wild turkey); UPI00016E2089 (A0A3B5K3K0): Takifugu rubripes (Japanese pufferfish) (Fugu rubripes); B5X3B0_SALSA: Salmo salar (Atlantic salmon); Q29RC5_DANRE: Danio rerio (Zebrafish) (Brachydanio rerio); UPI00017B3D0F: Tetraodon nigroviridis (Spotted green pufferfish) (Chelonodon nigroviridis); Q4S4J5_TETNG: Tetraodon nigroviridis (Spotted green pufferfish) (Chelonodon nigroviridis);

E6ZGP3_DICLA: Dicentrarchus labrax (European seabass) (Morone labrax); G3P4S1_GASAC: Gasterosteus aculeatus (Three-spined stickleback); B6VB99_SCOMX: Scophthalmus maximus (Turbot) (Psetta maxima); UPI00022AF5F0: Oreochromis niloticus (Nile tilapia) (Tilapia nilotica); D3TPD8_GLOMM: Glossina morsitans morsitans (Savannah tsetse fly); B4J2E5_DROGR: Drosophila grimshawi (Hawaiian fruit fly) (Idiomyia grimshawi); UPI00022B398F (A0A3P8P4B1): Astatotilapia calliptera (Eastern happy) (Chromis callipterus); B3M528_DROAN: Drosophila ananassae (Fruit fly); RAC1_DROME: Drosophila melanogaster (Fruit fly); F7AX30_HORSE: horse. Source: http://genetics.bwh.harvard.edu/pph2/index.shtml.

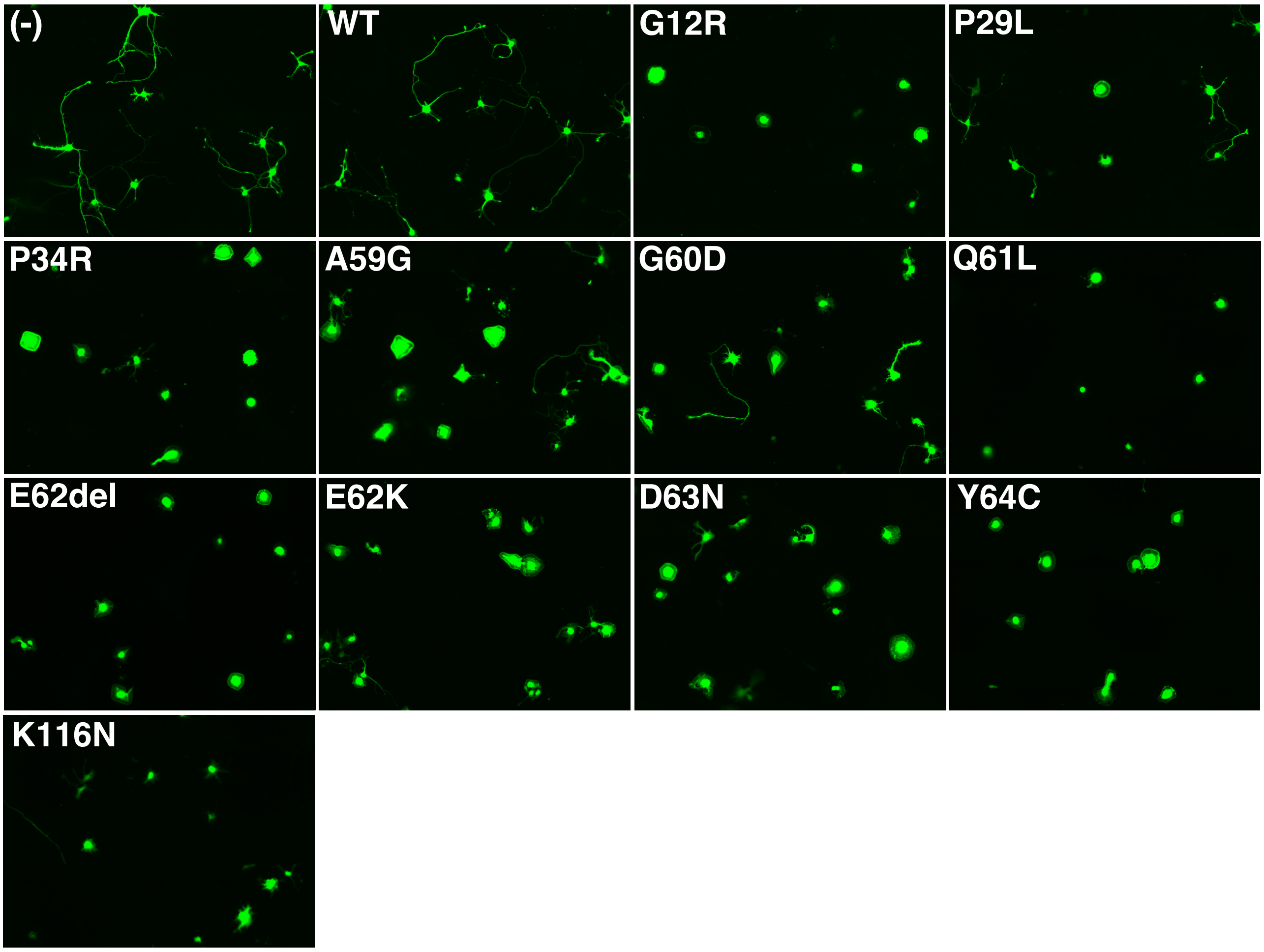

Supplementary Figure 5. Effects of the disease-causative 11 RAC3 variants on neuronal morphology *in vitro*. Hippocampal neurons dissociated at E16 were co-electroporated with pCAG-EGFP (0.1 μg) together with pCAG-Myc (-), pCAG-Myc-RAC3 (WT), -G12R, -P29L, -P34R, -A59G, -G60D, -Q61L, -E62del, -E62K, -D63N, -Y64C, and -K116N (0.3 μg each) and were cultured *in vitro* for three days. Control (-) and WT-expressing neurons began to extend an axon (the longest neurite) and dendrites. Although growth of axon and dendrites was severely suppressed in cells expressing the 11 variants, the ratio of round cells was dependent on the variation types, which affects the quantification analyses in Fig. 2.

(A)

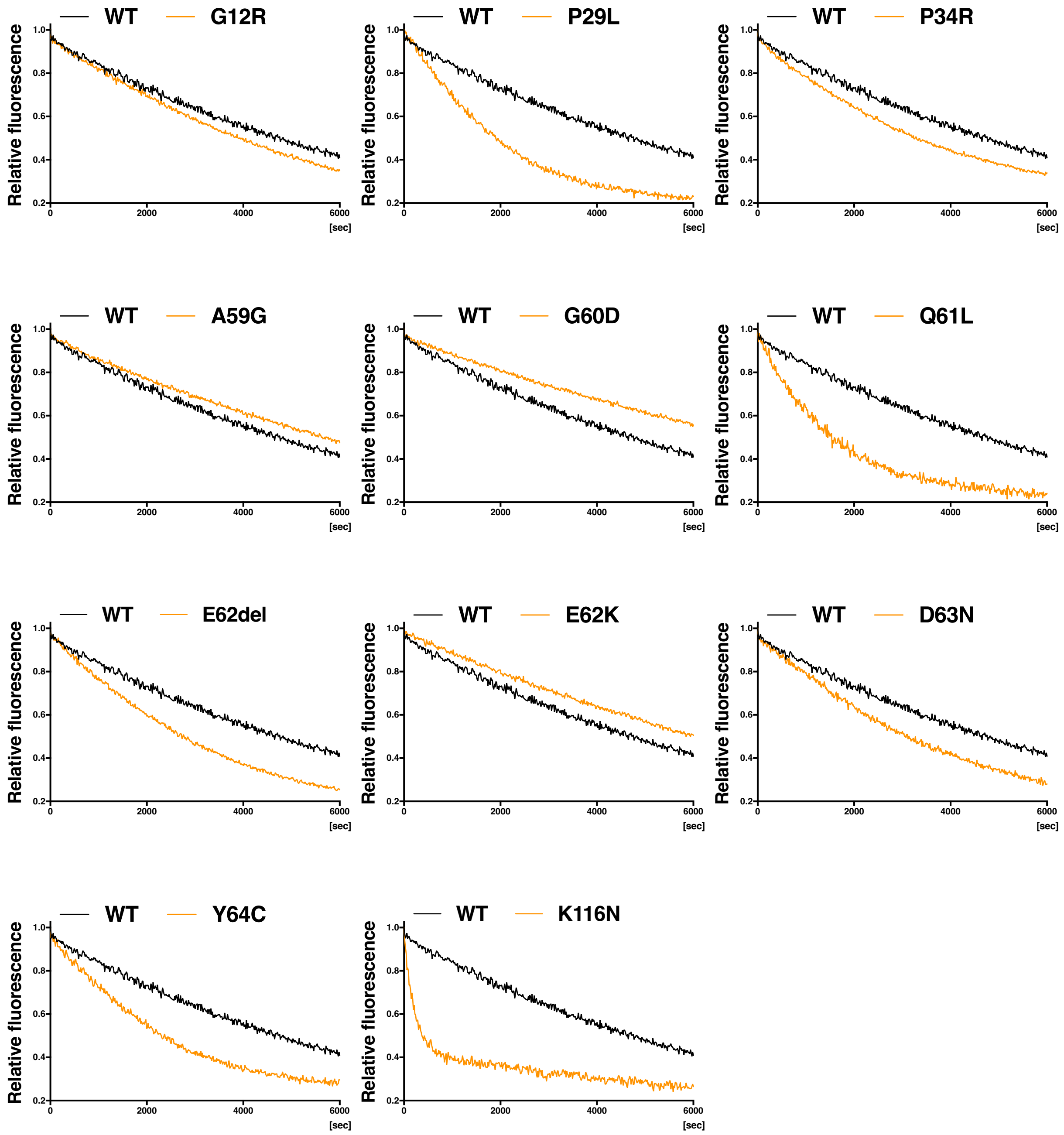

(B)

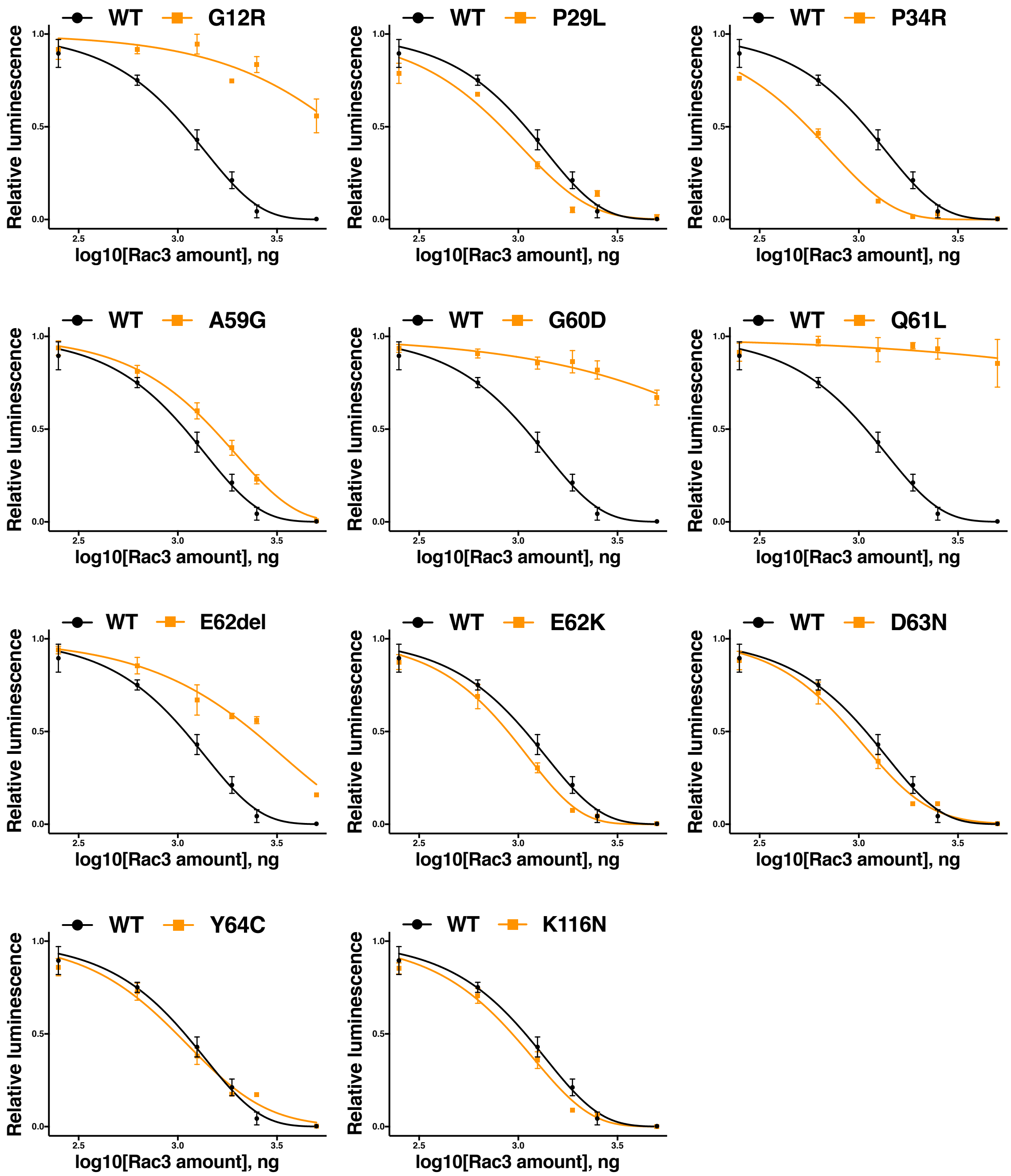

Supplementary Figure 6. Characterization of activation state of the disease-causative 11 RAC3 variants *in vitro*. (A) Raw data of GDP/GDP-exchange activity. The release of a fluorescently labelled GDP (^{mant}GDP) from each variant was measured as described in the "Materials and methods" section and compared to that of WT. The data were quantified and shown in Fig. 3A. **(B)** Raw data of intrinsic GTP-hydrolysis activity. The activity was measured as described in the "Materials and Methods" section. The data were quantified and shown in Fig. 3B.

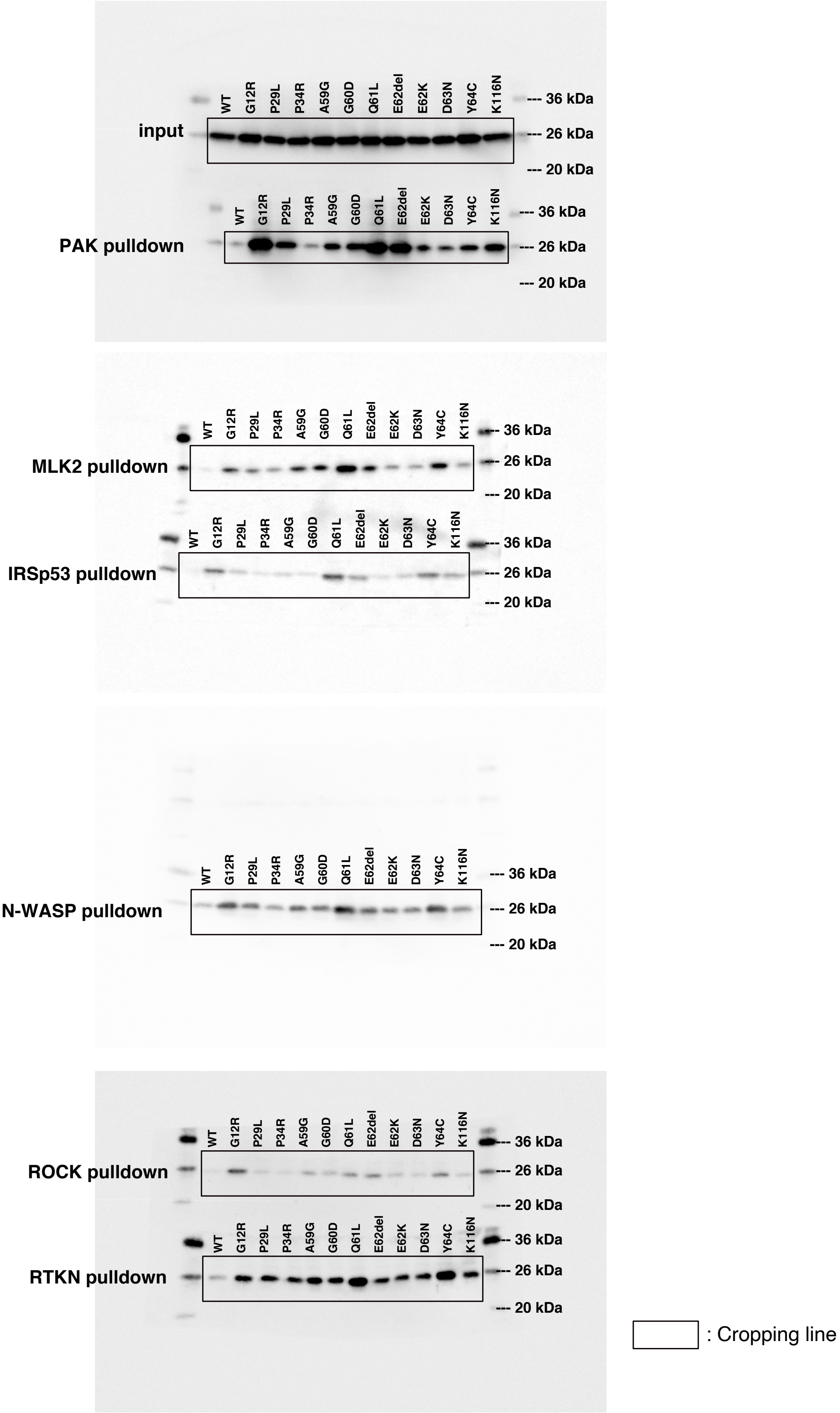

Supplementary Figure 7. Uncropped blotting data of the pull-down assay. The data in Fig. 4A were prepared by cropping of the original blots. The band intensity was measured by ImageJ software.

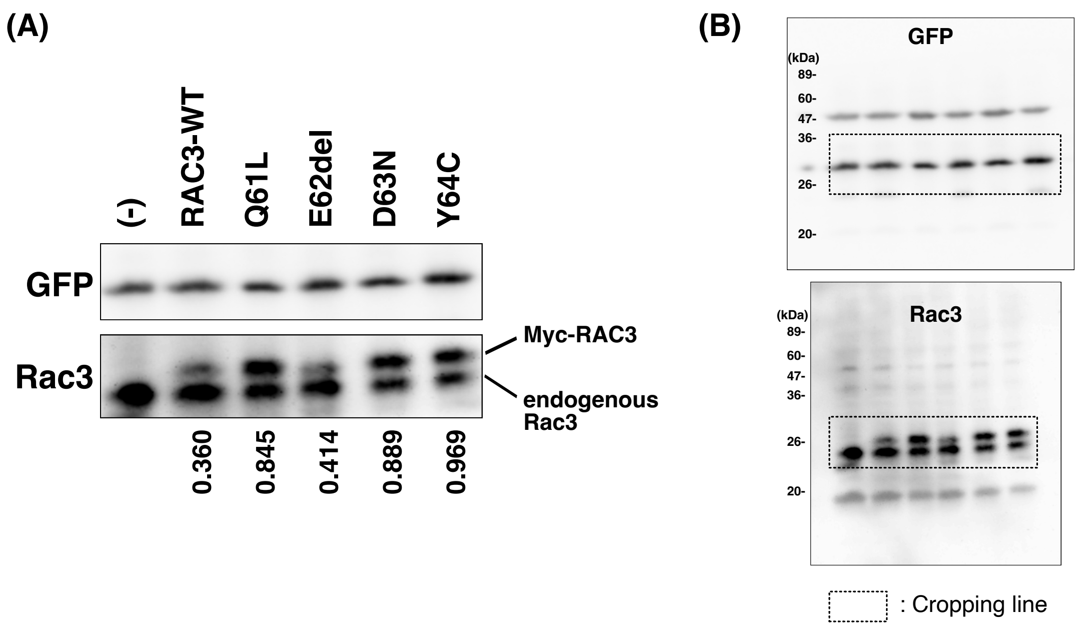

Supplementary Figure 8. Expression of the disease-causative variants in the Switch II region in cortical neurons with migration defects. (A) pCAG-EGFP (0.5 μg) was co-electroporated *in utero* with pCAG-Myc (-), pCAG-Myc-RAC3 (WT), -RAC3-Q61L, -E62del, -D63N, or -Y64C (0.1 μg each) into the VZ progenitor cells at E14.5. Cortical regions where plasmids were electroporated were separated at E16, and the whole lysates were subjected to western blotting. Endogenous Rac3 and overexpressed RAC3 proteins were visualized with anti-Rac3. Relative band intensities of Myc-RAC3 proteins were calculated with ImageJ software based on densitometry, and normalized against endogenous Rac3. The data shown are representative of three independent experiments. Note that glial cells are hardly generated at E16. **(B)** Uncropped western blot images presented in the Supplementary Fig. 8A.

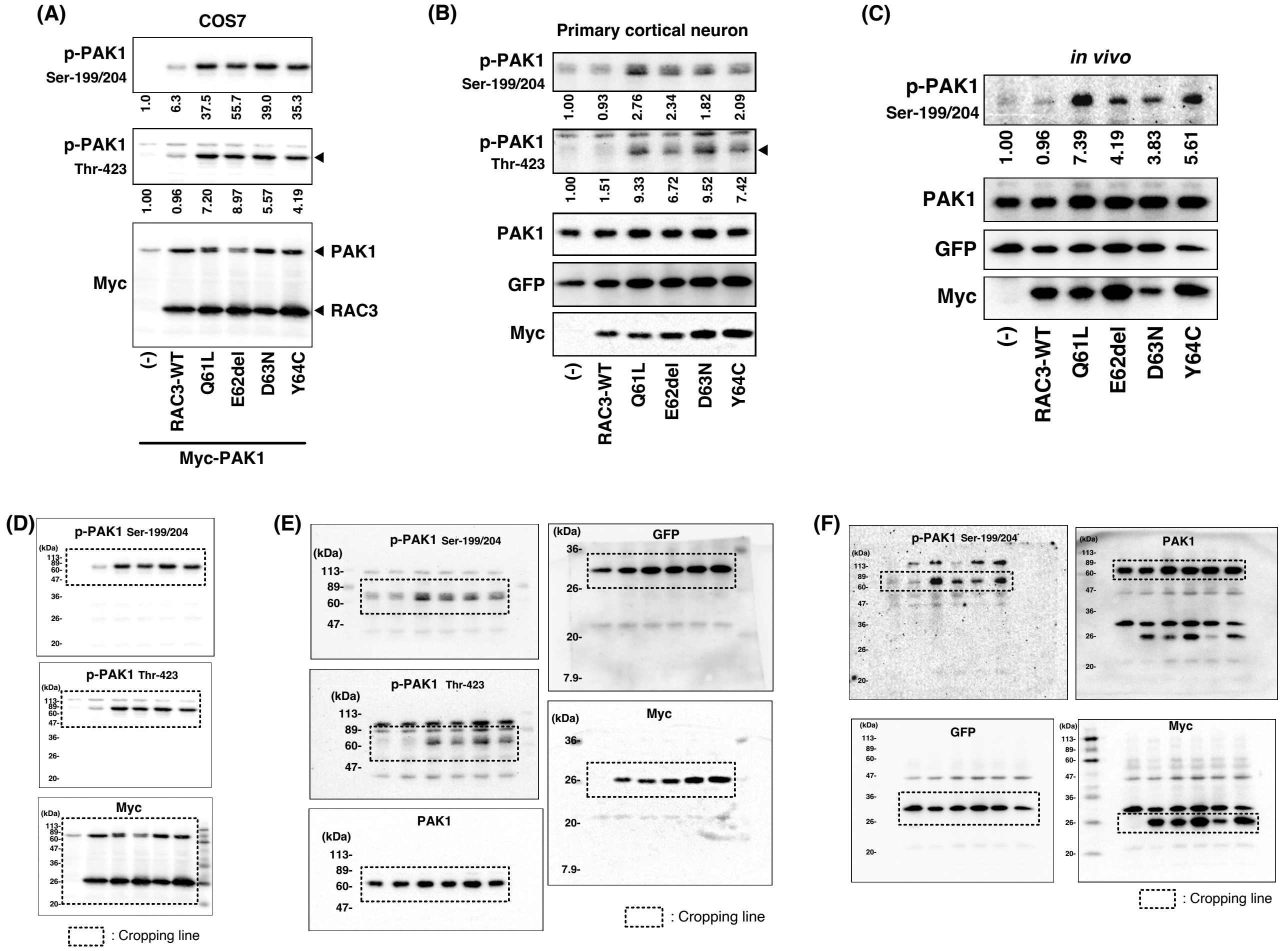

Supplementary Figure 9. Activation of PAK1 by the disease-causative variants in the Switch II region. **(A)** Activation of exogenous PAK1 in COS7 cells. Cells were transfected with pCAG-Myc-PAK1 (0.1 µg) together with pCAG-Myc (-), pCAG-Myc-RAC3 (WT), -Q61L, -E62del, -D63N, or -Y64C (0.1 µg each). After 24h, cell lysates were prepared and subjected to western blot analyses. Activated Myc-PAK1 was detected by anti-phospho-Ser199/204-PAK1 or anti-phospho-Thr423-PAK1. Myc-PAK1 and Myc-RAC3 were detected with anti-Myc. Relative band intensities of activated PAK1 were calculated with ImageJ software based on densitometry, and normalized against Myc-PAK1. **(B)** Activation of endogenous PAK1 in primary cortical neurons. Dissociated neurons from E16 mice were transfected with pCAG-GFP (0.5 µg) together with pCAG-Myc (-), pCAG-Myc-RAC3 (WT), -Q61L, -E62del, -D63N, or -Y64C (2.0 µg each). After cell lysates were prepared at 3 div, activated endogenous PAK1 was detected as in (A). Endogenous PAK1, GFP, and Myc-RAC3 were detected with anti-PAK1, anti-GFP, and anti-Myc, respectively. The data shown are representatives of three independent experiments. Relative band intensities of endogenous activated PAK1 were calculated with ImageJ software based on densitometry, and normalized against endogenous PAK1. **(C)** Activation of endogenous PAK1 in cortical tissue. pCAG-EGFP (0.5 µg) was co-electroporated *in*

utero with pCAG-Myc (-), pCAG-Myc-RAC3 (WT), -RAC3-Q61L, -E62del, -D63N, or -Y64C (0.2 µg each) into the VZ progenitor cells at E14.5. Cortical regions where plasmids were electroporated were separated at E16, and the whole lysates were subjected to western blotting. These lysates were prepared from different mouse (10 samples each). Endogens activated PAK1 was visualized with anti-phospho-Ser199/204-PAK1. The blot was then reprobed for endogenous PAK1, GFP, and Myc. Relative band intensities of endogenous activated PAK1 were calculated as in (B). **(D - F)** Uncropped western blot images presented in Supplementary Fig. 9A - C.

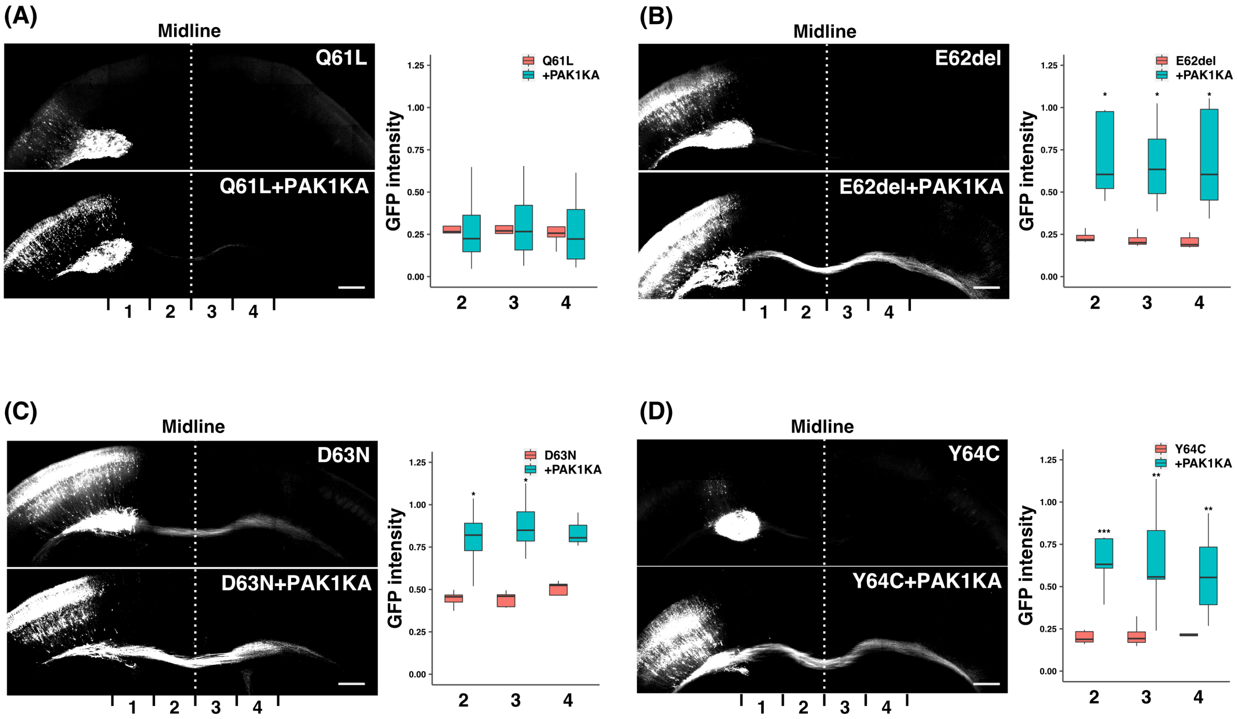

Supplementary Figure 10. Rescue effects by a dominantly negative PAK1 on axon growth defects caused by the four variants in the Switch II region. pCAG-Myc-RAC3-Q61L **(A)**, E62del **(B)**, -D63N **(C)**, or -Y64C **(D)** (0.1 µg each) was co-electroporated with pCAG-EGFP (0.5 µg) together with pCAG-Flag vector (1.0 µg, control) (*upper*) or pCAG-Flag-PAK1KA (1.0 µg) (*lower*). Analyses were done at P7 as in Fig. 8. Scale bars, 500 µm. The GFP intensity of the callosal axon was measured in different regions (bins 1 - 4), and then the relative intensities of bins were normalized with bin 1 as 1.0. Number of replicates, N ≧ 4. The significance of difference between control and each rescue condition was determined using Welch's t test. (A, bin 2) Q61L vs. +PAK1KA, p = 0.9833. (A, bin 3) Q61L vs. +PAK1KA, p = 0.8981. (A, bin 4) Q61L vs. +PAK1KA, p = 0.9638. (B, bin 2) E62del vs. +PAK1KA, p = 0.01392. (B, bin 3) E62del vs. +PAK1KA, p =

0.03876. (B, bin 4) E62del vs. +PAK1KA, $p = 0.02716$. (C, bin 2) D63N vs. +PAK1KA, $p = 0.0421$. (C, bin 3) D63N vs. +PAK1KA, $p = 0.02379$. (C, bin 4) D63N vs. +PAK1KA, $p = 0.08895$. (D, bin 2) Y64C vs. +PAK1KA, $p = 0.0006848$. (D, bin 3) Y64C vs. +PAK1KA, $p = 0.007151$. (D, bin 4) Y64C vs. +PAK1KA, $p = 0.007304$. ***$p < 0.001$, **$p < 0.01$, *$p < 0.05$.

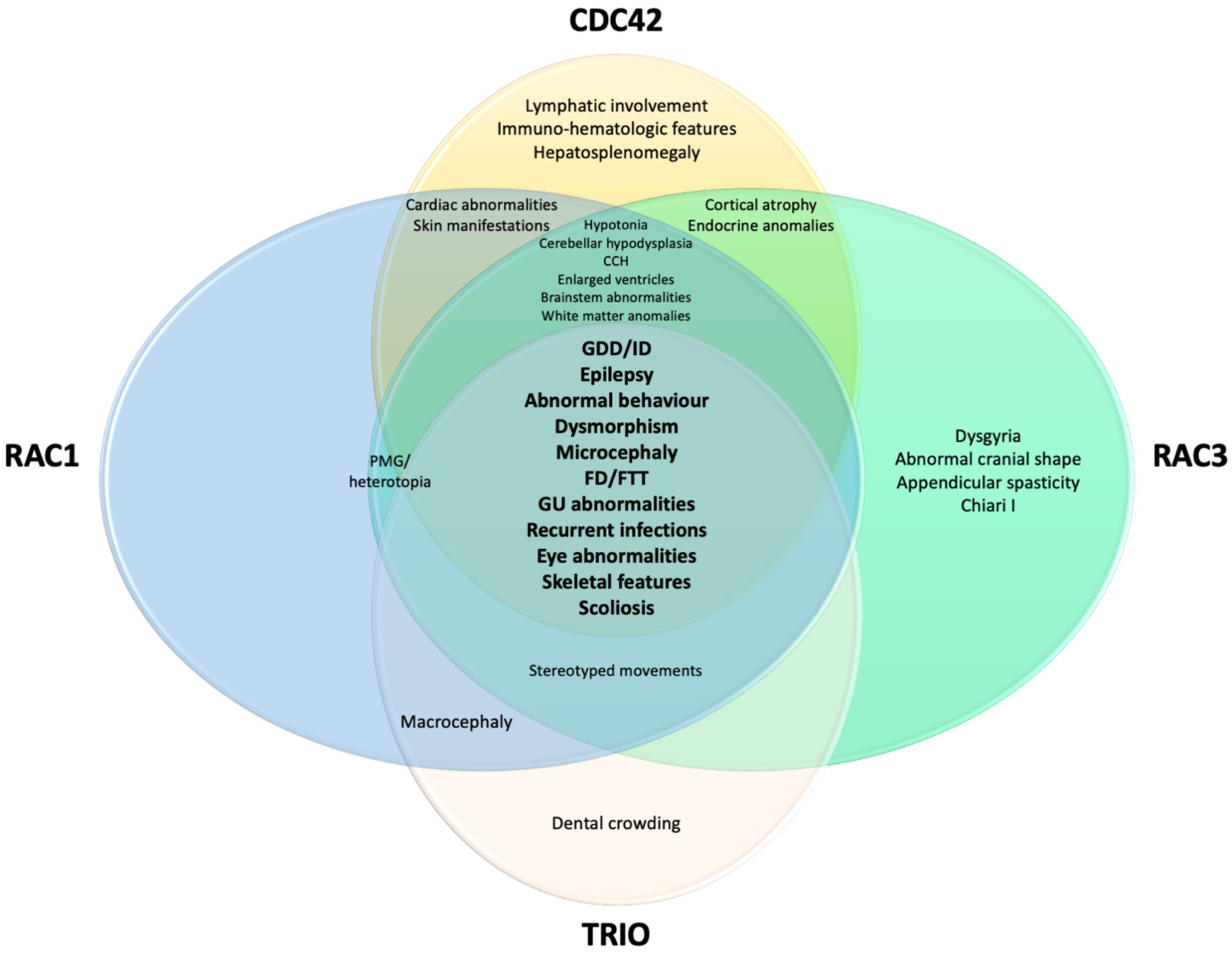

Supplementary Figure 11. Clinical overlap between Rho GTPase related disorders. Overlapping phenotypic features associated with *RAC1* (RAS-related c3 botulinum toxin substrate 1 - OMIM *602048; mental retardation, autosomal dominant 48 - OMIM #617751), *CDC42* (CELL DIVISION CYCLE 42 - OMIM *116952; TAKENOUCHI-KOSAKI SYNDROME - OMIM #616737), *RAC3* (RAS-related c3 botulinum toxin substrate 3 - OMIM *602050; neurodevelopmental disorder with structural brain anomalies and dysmorphic facies - OMIM #618577), and *TRIO* (Triple functional domain - * 601893; intellectual developmental disorder, autosomal dominant 44, with microcephaly or macrocephaly; OMIM #617061 and

#618825). Abbreviations: CCH = corpus callosum hypoplasia; FD = feeding difficulties; FTT = failure to thrive; GDD = global developmental delay; GU = genitourinary; ID = intellectual disability; PMG = polymicrogyria.

4. Supplementary Videos

Supplementary videos 1 and 2. Detailed neurological phenotype. Patient #9 at the age of two years shows generalized hypotonia, characteristic stereotypical movements of mouth and upper limbs (upward and forward extension, or stereotyped flexion of one limb at a time), hand mouthing, stereotyped deep breathing, and frequent eyelid clonus, and stereotyped guttural sounds.

Supplementary videos 3-7. Time-lapse imaging data. Cell migration was monitored from E16 for 15 h in the upper IZ and lower CP. Supplementary video 3: Time-lapse imaging of wild type RAC3-expressing cortical neurons. Supplementary video 4: Time-lapse imaging of RAC3-E62del-expressing cortical neurons. Supplementary video 5: Time-lapse imaging of RAC3-D63N-expressing cortical neurons. Supplementary video 6: Time-lapse imaging of RAC3-Q61L-expressing cortical neurons. Supplementary video 7: Time-lapse imaging of RAC3-Y64C-expressing cortical neurons.

5. Supplementary References

Aspromonte MC, Bellini M, Gasparini A, Carraro M, Bettella E, Polli R, Cesca F, Bigoni S, Boni S, Carlet O, Negrin S, Mammi I, Milani D, Peron A, Sartori S, Toldo I, Soli F, Turolla L, Stanzial F, Benedicenti F, Marino-Buslje C, Tosatto SCE, Murgia A, Leonardi E. Characterization of intellectual disability and autism comorbidity through gene panel sequencing. Hum Mutat. 2019 Sep;40(9):1346-1363. doi: 10.1002/humu.23822. Epub 2019 Aug 2. Erratum in: Hum Mutat. 2020 Jun;41(6):1183.

Bowling KM, Thompson ML, Amaral MD, Finnila CR, Hiatt SM, Engel KL, Cochran JN, Brothers KB, East KM, Gray DE, Kelley WV, Lamb NE, Lose EJ, Rich CA, Simmons S, Whittle JS, Weaver BT, Nesmith AS, Myers RM, Barsh GS, Bebin EM, Cooper GM. Genomic diagnosis for children with intellectual disability and/or developmental delay. Genome Med. 2017 May 30;9(1):43. doi: 10.1186/s13073-017-0433-1.

Cox DM, Butler MG. The 15q11.2 BP1-BP2 microdeletion syndrome: a review. Int J Mol Sci. 2015 Feb 13;16(2):4068-82. doi: 10.3390/ijms16024068. PMID: 25689425; PMCID: PMC4346944.

DePristo MA, Banks E, Poplin R, et al. A framework for variation discovery and genotyping using next-generation DNA sequencing data. Nat Genet. 2011;43:491–8.

Fromer M, Moran JL, Chambert K, et al. Discovery and statistical genotyping of copy-number variation from whole-exome sequencing depth. *Am J Hum Genet*. 2012;91(4):597-607.

Harripaul R, Noor A, Ayub M, Vincent JB. The Use of Next-Generation Sequencing for Research and Diagnostics for Intellectual Disability. Cold Spring Harb Perspect Med. 2017 Mar 1;7(3):a026864. doi: 10.1101/cshperspect.a026864. PMID: 28250017; PMCID: PMC5334248.

Li H, Durbin R. Fast and accurate short read alignment with Burrows-Wheeler transform. Bioinformatics. 2009;25:1754–60.

McKenna A, Hanna M, Banks E, et al. The Genome Analysis Toolkit: a MapReduce framework for analyzing next-generation DNA sequencing data. Genome Res. 2010;20:1297–303.

Murdock DR, Dai H, Burrage LC, Rosenfeld JA, Ketkar S, Müller MF, Yépez VA, Gagneur J, Liu P, Chen S, Jain M, Zapata G, Bacino CA, Chao HT, Moretti P, Craigen WJ, Hanchard NA; Undiagnosed Diseases Network, Lee B. Transcriptome-directed analysis for Mendelian disease

diagnosis overcomes limitations of conventional genomic testing. J Clin Invest. 2021 Jan 4;131(1):e141500. doi: 10.1172/JCI141500.

Rafi SK, Butler MG. The 15q11.2 BP1-BP2 Microdeletion (Burnside-Butler) Syndrome: In Silico Analyses of the Four Coding Genes Reveal Functional Associations with Neurodevelopmental Phenotypes. Int J Mol Sci. 2020 May 6;21(9):3296. doi: 10.3390/ijms21093296.

Redon R, Carter NP. Comparative genomic hybridization: microarray design and data interpretation. Methods Mol Biol. 2009;529:37–49.

Tarailo-Graovac M, Shyr C, Ross CJ, Horvath GA, Salvarinova R, Ye XC, Zhang LH, Bhavsar AP, Lee JJ, Drögemöller BI, Abdelsayed M, Alfadhel M, Armstrong L, Baumgartner MR, Burda P, Connolly MB, Cameron J, Demos M, Dewan T, Dionne J, Evans AM, Friedman JM, Garber I, Lewis S, Ling J, Mandal R, Mattman A, McKinnon M, Michoulas A, Metzger D, Ogunbayo OA, Rakic B, Rozmus J, Ruben P, Sayson B, Santra S, Schultz KR, Selby K, Shekel P, Sirrs S, Skrypnyk C, Superti-Furga A, Turvey SE, Van Allen MI, Wishart D, Wu J, Wu J, Zafeiriou D, Kluijtmans L, Wevers RA, Eydoux P, Lehman AM, Vallance H, Stockler-Ipsiroglu S, Sinclair G, Wasserman WW, van Karnebeek CD. Exome Sequencing and the Management of Neurometabolic Disorders. N Engl J Med. 2016 Jun 9;374(23):2246-55. doi: 10.1056/NEJMoa1515792.

Wang K, Li M, Hakonarson H. ANNOVAR: functional annotation of genetic variants from high-throughput sequencing data. Nucleic Acids Res. 2010;38:e164.

6. Supplementary Affiliations

Undiagnosed Disease Network

Gabrielle Brown,[1] Manish J. Butte,[1] Esteban C. Dell'Angelica,[1,2] Naghmeh Dorrani,[1,3] Emilie D. Douine,[1,2] Brent L. Fogel,[1,4] Irma Gutierrez,[1,2] Alden Huang,[1] Deborah Krakow,[1,5] Hane Lee,[1,6] Sandra K. Loo,[1] Bryan C. Mak,[1,2] Martin G. Martin,[1] Julian A. Martínez-Agosto,[1] Elisabeth McGee,[1] Stanley F. Nelson,[1] Shirley Nieves-Rodriguez,[1] Christina G.S. Palmer,[1] Jeanette C. Papp,[1,2] Neil H. Parker,[1,7] Genecee Renteria,[1] Janet S. Sinsheimer,[1,8] Jijun Wan,[1] Lee-kai Wang[1,9] and Katherine Wesseling Perry[1]

1 UCLA, Los Angeles, CA, USA

2 Department of Human Genetics, David Geffen School of Medicine at the University of California, Los Angeles

3 David Geffen School of Medicine, Department of Pediatrics

4 UCLA Departments of Neurology and Human Genetics, UCLA Clinical Neurogenomics Research Center

5 Department of Human Genetics, Department of Pediatrics, Department of Obstetrics and Gynecology, Department of Orthopaedic Surgery

6 Department of Pathology and Laboratory Medicine, Department of Human Genetics

7 David Geffen School of Medicine, Ronald Reagan Hospital

8 Departments of Human Genetics, Biomathematics, Biostatistics UCLA

9 Institute for Precision Health, David Geffen School of Medicine at University of California at Los Angeles

Role in UDN

Research patient navigator/coordinator (Gabrielle Brown); Co-Investigator (Manish J. Butte, Esteban C. Dell'Angelica, Brent L. Fogel, Deborah Krakow, Sandra K. Loo, Martin G. Martin, Jeanette C. Papp, Neil H. Parker, Janet S. Sinsheimer); Site coordinator and genetic counselor (Naghmeh Dorrani, Bryan C. Mak); Research Assistant (Emilie D. Douine, Genecee Renteria, Jijun Wan); Study Coordinator (Irma Gutierrez); Sequencing interpretation (Alden Huang, Hane Lee, Lee-kai Wang); PI (Julian A. Martínez-Agosto, Stanley

F. Nelson, Christina G.S. Palmer); Research Nurse (Elisabeth McGee); Graduate Student Researcher (Shirley Nieves-Rodriguez); Clinician (Katherine Wesseling Perry).

Telethon Undiagnosed Diseases Program

Vincenzo Nigro,[1,2] Nicola Brunetti-Pierri,[2,3] Giorgio Casari,[2,4] Gerarda Cappuccio,[2,3] Annalaura Torella,[1,2] Michele Pinelli,[2,3] Francesco Musacchia,[2] Margherita Mutarelli,[2] Diego Carrella,[2] Giuseppina Vitiello,[2,5] Valeria Capra,[6] Giancarlo Parenti,[2,3] Vincenzo Leuzzi,[7] Angelo Selicorni,[8] Silvia Maitz,[9] Sandro Banfi,[1,2] Marcella Zollino,[10] Mario Montomoli,[11] Donatelli Milani,[12] Corrado Romano,[13] Albina Tummolo,[14] Daniele De Brasi,[15] Antonietta Coppola,[16] Claudia Santoro,[17] Angela Peron,[18,19] Chiara Pantaleoni,[20] Raffaele Castello[2] and Stefano D'Arrigo[20]

1 Department of Precision Medicine, University of Campania Luigi Vanvitelli, Naples, Italy;

2 Telethon Institute of Genetics and Medicine, Pozzuoli, Italy;

3 Department of Translational Medicine, Federico II University, Naples, Italy;

4 Vita Salute San Raffaele University, Milan, Italy;

5 Department of Translational Medicine, Section of Pediatrics, Federico II University, Naples, Italy;

6 Neuroscience Department, Giannina Gaslini Institute, Genoa, Italy;

7 Department of Human Neuroscience, Sapienza University of Rome, Rome, Italy;

8 Department of Pediatrics, ASST Lariana, Sant'Anna Hospital, San Fermo della Battaglia, Como, Italy;

9 Fondazione MBBM, Monza, Italy;

10 Institute of Genomic Medicine, Catholic University, Gemelli Hospital Foundation, Rome, Italy;

11 Pediatric Neurology, Neurogenetics and Neurobiology Unit and Laboratories, Neuroscience Department, A Meyer Children's Hospital, University of Florence, Firenze, Italy;

12 Pediatric Highly Intensive Care Unit, Fondazione IRCCS Ca' Granda, Ospedale Maggiore Policlinico, Milan, Italy;

13 Associazione Oasi Maria SS Onlus, Troina, Italy;

14 Department of Metabolic Diseases, Clinical Genetics and Diabetology, Giovanni XXIII Children's Hospital, Bari, Italy;

15 Department of Pediatrics, AORN Santobono Pausilipon, Naples, Italy;

16 Department of Neuroscience, Reproductive and Odontostomatological Sciences, Epilepsy Centre, Federico II, University of Naples, Naples, Italy;

17 University of Campania Luigi Vanvitelli, Naples, Italy;

18 Dipartimento di Scienze della Salute, Neuropsichiatria Infantile-Centro Epilessia, Ospedale San Paolo, Università degli Studi di Milano, Milan, Italy;

19 Department of Pediatrics, Division of Medical Genetics, University of Utah School of Medicine, Salt Lake City, UT, USA;

20 Department of Developmental Neurology, Fondazione IRCCS Istituto Neurologico Carlo Besta, Milan, Italy.
