# Supplementary material for: Variant-specific changes in RAC3 function disrupt corticogenesis in neurodevelopmental phenotypes
Source: Brain. 2022 Jul 4;145(9):3308–27. doi: 10.1093/brain/awac106 (PMC9473360; doi:10.1093/brain/awac106)
Supplement: awac106_Supplementary_Data [file awac106_supplementary_data.zip › Supplementary_Table_2.pdf]

Supplementary Table 2. Clinical and neuroimaging features of individuals with *RAC3*-related disorder

	**Costain *et al.***	**Hiraide *et al.***	**This study**	**Total**
**Clinical features**				
Gender	4 M, 1 F	1 M	8 F, 2 M	**M:F = 0.77**
Global DD/ID	+ (5/5)	+	+ (10/10)	**16/16 (100%)**
Hypotonia	+ (5/5)	-	+ (10/10)	**15/16 (93.7%)**
Dysmorphic features	+ (5/5)	-	+ (8/10)	**13/16 (81.3%)**
Dysphagia	+ (4/5)	+	+ (6/10)	**10/16 (62.5%)**
Failure to thrive	+ (4/5)	+	+ (6/10)	**10/16 (62.5%)**
Seizures	+ (2/5)	+	+ (5/10)	**7/16 (43.7%)**
Musculoskeletal features	+ (2/5)	-	+ (5/10)	**7/16 (43.7%)**
Dyspraxia	NA	NA	+ (4/10)	**4/10 (40%)**
Genitourinary abnormalities	+ (1/5)	+	+ (4/10)	**6/16 (37.5%)**
Abnormal cranial shape	+ (1/5)	-	+ (4/10)	**5/16 (31.2%)**
Respiratory problems	-	+	+ (4/10)	**5/16 (31.2%)**
Behavioral disorders	NA	-	+ (3/10)	**3/11 (27.3%)**
Stereotyped movements	NA	-	+ (3/10)	**3/11 (27.3%)**
Spasticity	-	+	+ (3/10)	**4/16 (25%)**
Eye abnormalities	-	-	+ (4/10)	**4/16 (25%)**
Endocrinological features	-	-	+ (3/10)	**3/16 (18.7%)**
Microcephaly	+ (1/5)	-	+ (1/10)	**2/16 (12.5%)**
Hearing loss	-	-	+ (1/10)	**1/16 (6.2%)**
**Neuroimaging features**				
CCH/CCA	+ (5/5)	+	+ (10/10)	16/16 (100%)
White matter thinning	+ (5/5)	+	+ (8/10)	14/16 (87.5%)
MCD	+ (5/5)	+	+ (6/10)	12/16 (75%)
Polymicrogyria	+ (3/5)	-	+ (5/10)	8/16 (50%)
Dysgyria	+ (2/5)	+	+ (6/10)	8/16 (50%)
Brainstem abnormalities	+ (3/5)	+	+ (4/10)	8/16 (50%)
Cerebellar dysplasia	-	+	+ (6/10)	7/16 (43.7%)
Gray matter heterotopia	+ (2/5)	-	+ (1/10)	3/16 (18.7%)

CCA= corpus callosum agenesis; CCH = corpus callosum hypoplasia; DD = developmental delay; F = female; ID = intellectual disability; M = male; MCD= Malformation of cortical development; NA = not available.
